# Automatic scoring of COVID-19 severity in X-ray imaging based on a novel deep learning workflow

**DOI:** 10.1038/s41598-022-15013-z

**Published:** 2022-07-27

**Authors:** Viacheslav V. Danilov, Diana Litmanovich, Alex Proutski, Alexander Kirpich, Dato Nefaridze, Alex Karpovsky, Yuriy Gankin

**Affiliations:** 1 Quantori, Cambridge, MA USA; 2 Politecnico di Milano, Milan, Italy; 3 Beth Israel Deaconess Medical Center, Boston, MA USA; 4 Georgia State University, Atlanta, GA USA; 5 Kanda Software, Newton, MA USA

**Keywords:** Applied mathematics, Computational science, Computer science, Information technology, Scientific data, Bacterial infection, Tuberculosis, Viral infection, Radiography, Medical imaging, Outcomes research

## Abstract

In this study, we propose a two-stage workflow used for the segmentation and scoring of lung diseases. The workflow inherits quantification, qualification, and visual assessment of lung diseases on X-ray images estimated by radiologists and clinicians. It requires the fulfillment of two core stages devoted to lung and disease segmentation as well as an additional post-processing stage devoted to scoring. The latter integrated block is utilized, mainly, for the estimation of segment scores and computes the overall severity score of a patient. The models of the proposed workflow were trained and tested on four publicly available X-ray datasets of COVID-19 patients and two X-ray datasets of patients with no pulmonary pathology. Based on a combined dataset consisting of 580 COVID-19 patients and 784 patients with no disorders, our best-performing algorithm is based on a combination of DeepLabV3 + , for lung segmentation, and MA-Net, for disease segmentation. The proposed algorithms’ mean absolute error (MAE) of 0.30 is significantly reduced in comparison to established COVID-19 algorithms; BS-net and COVID-Net-S, possessing MAEs of 2.52 and 1.83 respectively. Moreover, the proposed two-stage workflow was not only more accurate but also computationally efficient, it was approximately 11 times faster than the mentioned methods. In summary, we proposed an accurate, time-efficient, and versatile approach for segmentation and scoring of lung diseases illustrated for COVID-19 and with broader future applications for pneumonia, tuberculosis, pneumothorax, amongst others.

## Introduction

Despite originating in late 2019, the novel coronavirus SARS-COV-2 (COVID-19) continues to spread and evolve worldwide, placing a lasting and unprecedented strain on the global healthcare community^1–3^. The virus may present itself in both mild and severe forms and can, in both cases, progress into a form that requires hospitalization, with certain patients admitted to intensive care units (ICUs)^4,5^. The sustained pressure, coupled with the development of new variants, requires the investigation of all possible screening mechanisms that not only aid in diagnosing the presence of the infection but also its severity, in a timely manner^6,7^.

Critical to lifting the burden placed on the healthcare community is the necessity to determine the severity of the infection both quickly and efficiently, allowing for streamlined resource allocation as well as effective patient care. For patients admitted to the hospital with a suspected COVID-19 infection, radiographic imaging has become part of the standard diagnostic procedure, with chest X-rays (CXR) readily preferred to computed tomography (CT)^8–10^. The extent of the infection is inferred from the visual cues present in a patients’ CXR, such as ground-glass opacities and their geographic extent throughout the lungs^11^.

Determining the extent of the infection from CXRs is both a non-trivial and time-consuming task, necessitating the adoption of computer-aided clinical support tools. Utilization of artificial intelligence (AI) in the fight against COVID-19 has, thus far, focused largely on the identification of the infection and distinguishing it from other forms of pneumonia^12–18^. Despite this, limited attention has been given to the identification of the infection severity. To address this knowledge gap, we propose a novel two-stage disease scoring workflow based on image segmentation and multi-task learning. We validate the performance of our proposed workflow against the combined scoring of two expert radiologists. We note that this workflow is not limited to the study of COVID-19 and may be extended to evaluate the severity of other lung disorders.

To determine the most optimal workflow we evaluated nine state-of-the-art lung and disease segmentation networks (U-net^19^, U-net +  + ^20^, DeepLabV3^21^, DeepLabV3 + ^22^, FPN^23^, Linknet^24^, PSPNet^25^, PAN^26^, MA-Net^27^) and found the best performing configurations as determined by the combined accuracy and complexity. The latter is of particular importance as it allows the broader scientific community to adopt the determined hyper-parameters for further research, extending beyond the scope of this work. To study algorithm performance, we collected, cleaned, and pre-processed three lung segmentation datasets as well as four disease segmentation and scoring datasets acquired for COVID-19 and pneumonia-infected patients. The datasets are made publicly available^28,29^. We compared our results against two known tailor-made solutions, BS-net^30^ and COVID-Net-S^31^. The source code used for model development, tuning, training and testing, and the obtained segmentation models are also made publicly available^32^.

## Related work

Recent advances in medical imaging, with the added utility of portable X-ray machines, led to a rise in the adoption of CXRs when monitoring patients admitted to ICUs^33^. Furthermore, analysis of CXRs plays a crucial role in the diagnosis and progression of acute respiratory distress syndrome (ARDS) and lower-respiratory tract infection (LRI)^34,35^. Access to quantifiable methods for radiograph-based severity scoring can thus greatly assist in a patients’ risk stratification^36,37^.

Despite its potential advantages, investigation into severity scoring in radiograph images has thus far been limited. Sheshdari et al. developed a radiologic severity index (RSI) to predict a 30-day mortality after an LRI diagnosis^38^. RSI scores (0–72) were based on pulmonary infiltrates as well as the degree of their geographic involvement. To assist with the interpretation of CXRs, Taylor et al. developed a severity scoring tool for severe acute respiratory infections (SARI)^39^. The scoring tool made use of a 5-point system based on the extent of lung abnormalities, with resultant scores validated against those assigned by trained clinicians. In 2018, Warren et al., introduced the radiographic assessment of lung oedema (RALE) score to evaluate the degree of pulmonary oedema in ARDS^40^. The score (0–48) is calculated by dividing a radiograph image into quadrants with each quadrant scored on the extent of involvement (0–4; 0 meaning no involvement and 4 meaning 75% or more geographic involvement) and degree of opacification (1–3; hazy, intermediate, and dense). The method has shown diagnostic promise for the evaluation of ARDS in ICU admitted patients^41^ and has recently been extended to diagnose the severity of a patients’ COVID-19 infection^31,42–45^.

Wong et al. adapted RALE by assigning a score (0–4) to each lung based on the extent of lung involvement by consolidation or ground-glass opacity^44^. This methodology, though further adapted, has readily been applied when investigating the performance of deep learning models developed for severity detection^31,46,47^.

Adopting such scoring methodologies, Cohen et al., made use of seven non-COVID-19 datasets to pre-train a DenseNet model for feature extraction and task prediction^46^. Subsequently, a linear regression model was utilized to predict the score of each image. When evaluating the model performance against scoring performed by experts, the authors report an R-squared of 0.62 for the degree of opacity and 0.67 for the extent of involvement.

Extending upon the COVID-Net architecture^14^, Wong et al. propose COVID-Net-S to capture the severity of the COVID-19 infection^31^. By making use of 396 CXRs and the adapted RALE score, the authors report an R-squared score of 0.739 and 0.741 between predicted and expert scores for geographic involvement and opacity extent, respectively.

In 2020 Broghesi et al.^11^ introduced an alternative semi-quantitative score (0–18), namely the Brixia score. Here, each lung is divided into three regions with each region ranked (0–3) based on the extent of lung abnormalities. The Brixia score has shown promise when used to predict the risk of in-hospital mortality^48^ and the need for ventilatory support^49^. Subsequently, Signoroni et al.^30^ made use of the Brixia score to evaluate their end-to-end deep learning network when tasked with assessing the severity of a COVID-19 infection. By utilizing a dataset consisting of 5000 CXRs the authors reported mean absolute errors of 0.441 as compared to expert radiologists.

## Data

This section is devoted to the description of the data used to evaluate the proposed method. Since the proposed workflow is based on two independent stages, we adopted two different datasets for the training and testing of neural networks respectively.

### Stage I: lung segmentation dataset

The first stage of the proposed workflow is utilized for lung segmentation. Here, we collected and pre-processed three publicly available datasets, including the Darwin, Montgomery, and Shenzhen datasets^50–52^. These datasets include CXRs acquired for patients diagnosed with either COVID-19, pneumonia, or tuberculosis. It is worth noting that we utilize CXRs with different diagnoses solely for model training during Stage I. Since Stage I is tasked with lung segmentation, the use of images with different pathologies, differing in nature and disease patterns, acts as an augmentation technique and improves the generalization ability of studied networks. Table 1 contains a short description of these datasets and their split over the training, validation, and testing subsets.

**Table 1:** Description of the datasets used for lung segmentation.

Dataset	Source	Training	Validation	Testing	Total
Darwin	^50^	4884	611	611	6106/90%
Montgomery	^51^	110	14	14	138/2%
Shenzhen	^52^	452	57	57	566/8%
Total	–	5446/80%	682/10%	682/10%	6810/100%

The Darwin dataset images include most of the heart, revealing lung opacities behind the heart, which may be relevant for assessing the severity of viral pneumonia. The lower-most part of the lungs, where visible, is defined by the extent of the diaphragm. Where present and not obstructive to the distinguishability of the lungs, the diaphragm is included up until the lower-most visible part of the lungs. A key property of this dataset is that image resolutions, sources, and orientations vary across the dataset, with the smallest image being 156 × 156 pixels and the largest being 5600 × 4700 pixels. Furthermore, we include portable CXRs. Despite the latter being of significantly lower quality, such image variety allows for the improvement of the generalization ability of studied neural networks. It is worth noting that, initially, lung segmentations were performed by Darwin's Auto-Annotate AI and then adjusted and reviewed by human annotators.

**Figure 1:**
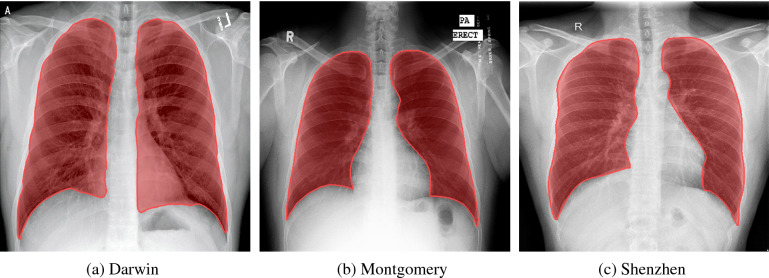
Examples of the collected chest X-ray images used for lung segmentation.

Both the Montgomery and Shenzhen datasets were published by the United States National Library of Medicine and consisted of posteroanterior chest X-ray images^53^. These images are available to foster research in computer-aided diagnosis of pulmonary diseases. The datasets were acquired from the Department of Health and Human Services (Maryland, USA) and Shenzhen №3 People's Hospital (Shenzhen, China). Both datasets contain normal and abnormal CXRs, with manifestations of tuberculosis, and include associated radiologist readings. Three examples of CXRs taken from all collected lung segmentation datasets are presented in Fig. 1.

### Stage II: disease segmentation and scoring dataset

Stage II of the proposed workflow is used for disease segmentation with subsequent scoring. Here, we collected and pre-processed four publicly available COVID-19 datasets including Actualmed COVID-19 Chest X-ray Dataset (ACCD)^54^, COVID-19 Radiography Database (CRD)^55^, COVID Chest X-Ray Dataset (CCXD)^56,57^, and Fig. 1 COVID Chest X-ray Dataset (FCXD)^58^. All those datasets include CXRs of subjects diagnosed with COVID-19 and were acquired across over 40 medical institutions and hospitals. In order to ensure the network’s generalization and distinctive abilities, we include subjects with no disease pathology, excluding any abnormalities and disorders. These subjects are represented by two datasets, namely Chest X-ray Normal Dataset (CXN)^59^ and RSNA Normal Dataset (RSNA)^60,61^. Both datasets were validated by two radiologists from our team, where they excluded images with indications of a pulmonary pathology. All healthy patients of these datasets are assigned a score of 0. Below, in Table 2, we summarize how both COVID-19 and normal datasets were split into training, validation, and testing subsets.

**Table 2:** Description of the datasets used for COVID-19 segmentation and scoring.

Dataset	Source	Diagnosis		Subset			Total
		COVID-19	Normal	Training	Validation	Testing	
ACCD	^54^	49	0	39	5	5	49/4%
CRD	^55^	104	0	83	10	11	104/8%
CCXD	^56,57^	399	0	319	40	40	399/29%
FCXD	^58^	28	0	22	3	3	28/2%
CXN	^59^	0	431	344	43	44	431/31%
RSNA	^60,61^	0	353	282	35	36	353/26%
Total	–	580/43%	784/57%	1089/80%	136/10%	139/10%	1364/100%

In order to achieve the ground truth segmentation and scores of the abnormal datasets and validate the normal datasets, two senior radiologists in our team, from the United States and Russia, annotated anteroposterior and posteroanterior radiographs. Each radiologist has more than ten years of experience and was active during the COVID-19 pandemic. The annotators labeled each dataset independently. Such a strategy, although time-consuming, allows us to get a consensus segmentation and severity score, which in turn helps us to determine the scoring ability of the proposed workflow when compared to the “gold standard” i.e. to the radiologist’s consensus.

Having analyzed the COVID-19 datasets, we summarized their main demographic characteristics in Table 3. Accordingly, subject ages fall between 36 and 70 years. Analysis of the age distribution shown in Appendix A (Figure A2), indicates that COVID-19 affects 91.6% of subjects aged from 25 to 80 years old, highlighting the severity of the global disease burden. We emphasize that the majority of the combined dataset records (53.7%) were acquired in European countries (Germany, Italy, Spain, and the United Kingdom). The distribution of the COVID-19 records by country is provided in Appendix A. We note that certain demographic information, such as age, gender, and the name of the collecting organization, is absent, in specific cases, due to the necessity to preserve patient anonymity.

**Table 3:** Demographic representation of the collected COVID-19 dataset.

Parameter	Absolute value	Relative value (%)
**Age**	53.5 ± 16.9	–
Male	52.6 ± 16.7	–
Female	55.0 ± 17.3	–
**Gender**		
Male	570	64
Female	322	36
**View**		
Anteroposterior	492	47
Posteroanterior	546	53
**Country (top-10)**		
Germany	169	19.6
Italy	165	19.1
Australia	84	9.7
China	77	8.9
Spain	69	8.0
United Kingdom	60	7.0
United States	40	4.6
Taiwan	18	2.1
South Korea	18	2.1
Iran	16	1.9

**Figure 2:**
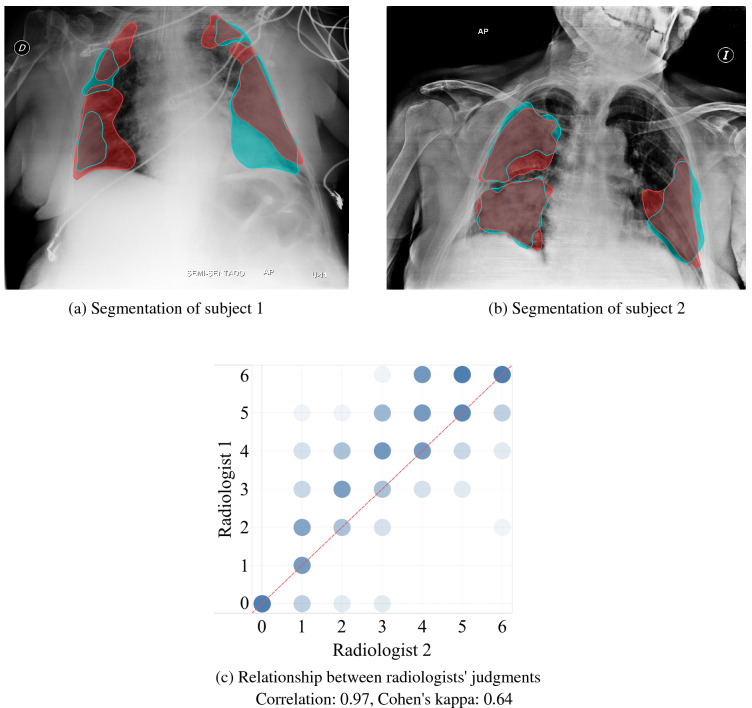
The comparison of segmentation and scoring between the two radiologists when the scoring was performed by both. The number of compared pairs of scores was 1085. Cyan and red masks were annotated by Radiologist 1 and Radiologist 2 respectively. The red dashed line indicates a perfect agreement between radiologists' judgments.

To evaluate the agreement of segmentation and scoring as determined by the two radiologists, their scores were compared pairwise for each CXR, as shown in Fig. 2. The visual summaries for 1085 pairs which were available for both radiologists are provided as a heatmap, Fig. 2c, where a darker color indicates a higher density of points and points on the red dashed line indicate perfect agreement. Most of the points are concentrated tightly around the dashed line with the only difference being that the first radiologist appeared to give slightly higher (one point) scores than the second one. For a more formal, numerical comparison between the judgment scores of two radiologists, the correlation coefficient (ρ = 0.97) and Cohen’s kappa statistic (κ = 0.64) were computed for all pairs of scores, where each element of the pair is the score of the corresponding radiologist for a given image. The computed correlation coefficient of 0.97 is close to 1 indicating a strong positive correlation and good agreement between the judgments. In the same way, the Cohen’s kappa value of 0.64 is interpreted as “substantial”^62^ and indicates a good agreement between the two judgments. Therefore, the radiologist’s scores were deemed to be robust measures with limited variability from radiologist to radiologist. These paired scores were then used to compute the “averaged” score from each pair, when available for both, and later used as a “gold standard” or a consensus for method evaluation and comparison. When only a single score was available from one radiologist, that score value was used as the “gold standard” which increased the total number of available scored images to 1364.

## Methods

**Figure 3:**
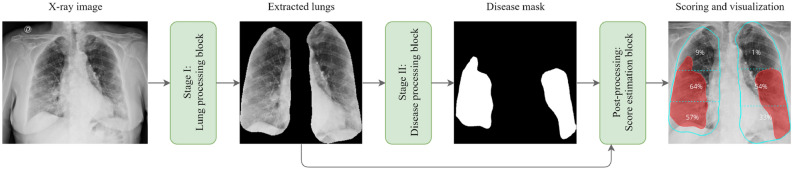
Schematic illustration of the proposed workflow which consists of three main stages: Stage I which pre-processes the lungs, Stage II which processes the disease areas, and the post-processing stage which performs the scoring.

In this section, we provide a detailed explanation of the proposed workflow, including both stages and the post-processing block used for the final scoring estimation. Additionally, we describe how the studied networks are hyper-tuned, trained, and how their performance is estimated in terms of accuracy, error rate, complexity, and processing speed. The proposed workflow inherits the quantification and qualification of lung diseases (scoring and decision-making) from expert radiologists, and fulfills the following processing steps, as shown in Fig. 3:

- Lung segmentation: pixel-level localization of the lungs and removal of unnecessary areas;
- Disease segmentation: pixel-level localization of the infected area of the lungs;
- Severity scoring: quantification and qualification of the infected area of the lungs.

When developing the proposed workflow, we tried to mimic the visual inspection and evaluation of X-ray images performed by radiologists and clinicians, when assessing and estimating the extent of lung disorders. We relied on input from expert radiologists to further understand the nature of this visual assessment. Here, an expert would first assess an image based on whether a suspected infection is present and further study the degree of pulmonary involvement on a 0 to 6 grading scale (low to high).

### Stage I: lung segmentation

**Figure 4:**
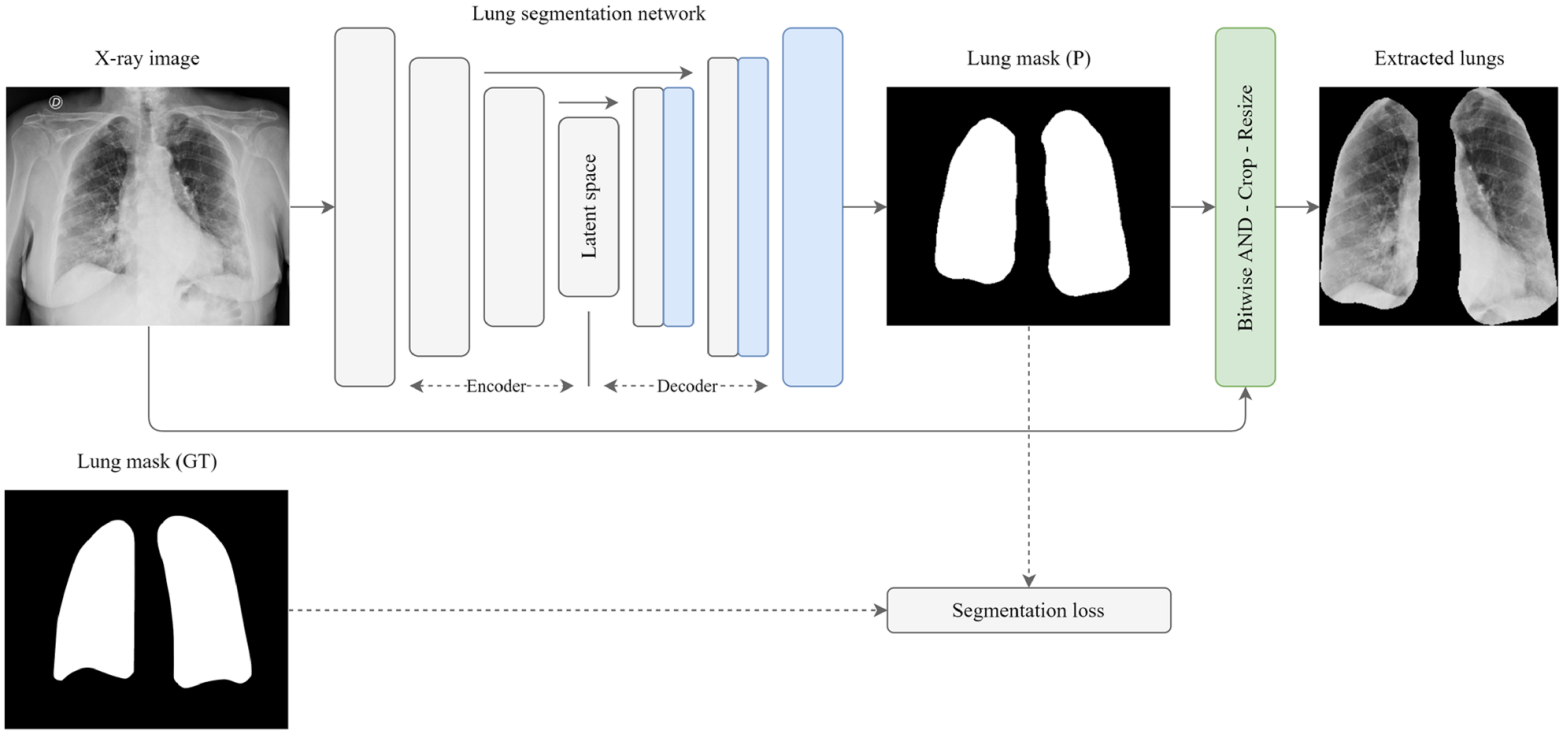
Lung segmentation stage (Stage I). The prefixes “P” and “GT” stand for prediction and ground truth, respectively.

In Stage I, we employ and compare different segmentation neural networks, including nine state-of-the-art solutions^19–27^. Lung segmentation is followed by a processing block used to exclude any unnecessary areas. As shown in Fig. 4, this block applies the bitwise AND operator (calculating the conjunction of pixels in both images) on the source image, using the predicted binary mask. Once this operation is performed, an image with the extracted lungs is cropped by the lung-bounding box and is then resized. The latter operation is useful during training because if the ground truth is not large, the training signal magnitude will be small. Such an issue is similar to the gradient vanishing problem and may lead to the general inability of a network, with many layers, to learn on a given dataset or prematurely converge to a poor solution. In such a scenario, due to a weak magnitude, the gradient may diminish dramatically as it is propagated backward through the network. The error may be very small by the time it reaches layers close to the input of the network and thus may have very little effect. To circumvent this, Stage I excludes any informative regions beyond the lungs with the aim of increasing the gradient magnitude during back-propagation.

### Stage II: disease segmentation

**Figure 5:**
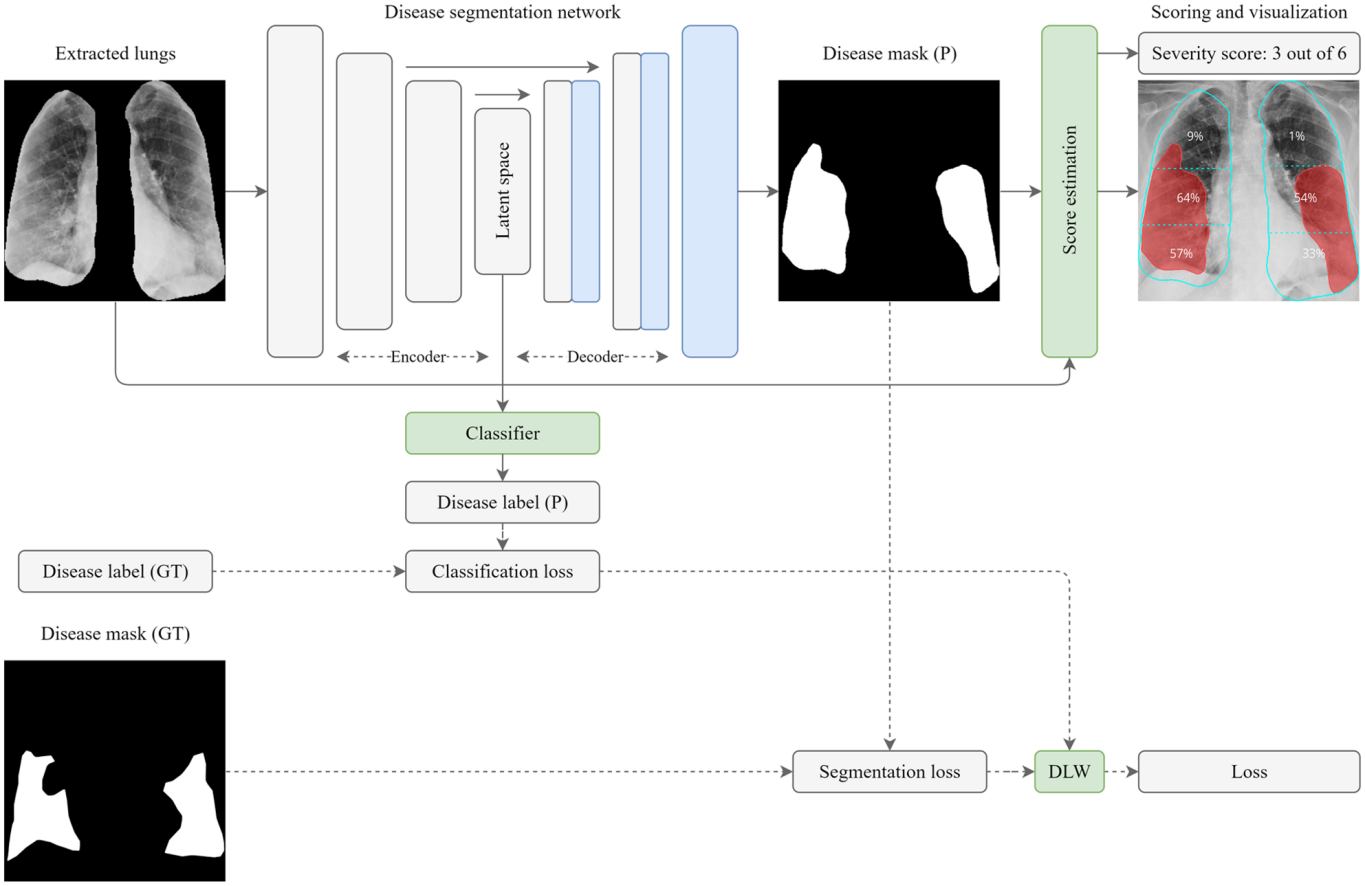
Disease segmentation stage (Stage II) and score estimation. The prefixes “P” and “GT” stand for prediction and ground truth, respectively.

Stage II is concerned with the application of Multi-Task Learning (MTL)^63,64^, as opposed to the Single-Task Learning (STL) of the first stage. The proposed MTL disease segmentation model, reflected in Fig. 5, has two branches, namely classification and segmentation. The need for the classification branch stems from the limited size of the training subset and is used to predict the class of an input image in order to regularize the shared encoder and impose additional constraints on its layers. MTL aims to learn multiple different tasks simultaneously while maximizing the performance across all tasks. The workflow of Stage II is based on the Hard Parameter Sharing of the MTL approach because of the need to simultaneously predict the disease label (classification) and the affected lung area (segmentation). In the proposed MTL workflow, the encoder plays the role of a Convolutional Neural Network (CNN) feature extractor, while the head of the classification branch (classifier) is used for making class predictions. The classifier predictions, thus, adopt the role of a regularizer and are used to refine segmentation predictions. Taking into account the highly confident classification outputs, the model refines the output of the segmentation branch i.e. the segmentation mask according to Eqs. (1) and (2). The classification branch outputs a probability of an image being a normal case (probability tends to 0) or being a disease case (probability tends to 1). The refined segmentation mask ${f}_{ref}^{seg}\left(x\right)$ is computed in the following manner:

1 $${f}_{ref}^{seg}\left(x\right)= {f}_{raw}^{seg}\left(x\right) \odot {f}_{bin}^{cls}\left(x\right)$$

2 $${f}_{bin}^{cls}\left(x\right)=\left\{\begin{array}{c}1,  \quad \quad if\, {f}_{raw}^{cls}\left(x\right) \ge T\\ 0,   \quad \quad if \,{f}_{raw}^{cls}\left(x\right) <T\end{array}\right.$$

where $x$ is an input image, ${f}_{raw}^{seg}\left(x\right)$ is a raw probability map made by the segmentation branch, ${f}_{raw}^{cls}\left(x\right)$ is the probability of an image being normal or disease-infected, made by the classification branch, ${f}_{bin}^{cls}\left(x\right)$ is a binarized ${f}_{raw}^{cls}\left(x\right)$, $\odot$ is the element-wise multiplication, $T$ is a threshold equal to 0.5 in our study.

We have found, experimentally, that the adopted regularization helps the proposed workflow decrease the false-positive rate on the segmentation branch. For the regulizer architecture, we used a reliable set of layers with proven stability on classification tasks and includes an adaptive two-dimensional pooling layer, flatten layer, dropout layer with a dropout rate of 0.20, and a dense one-neuron layer with a sigmoid activation function.

It has been shown that MTL models can often improve accuracy relative to independent STL models^65–67^. However, even when the average task accuracy improves, individual tasks may experience negative transfer where MTL model’s predictions are worse than that of STL models. To avoid this, we integrate and utilize Dynamic Loss Weighting (DLW)^68^ of the MTL model which combines and expands upon ideas from Reinforced MTL^69^ and Gradient Normalization for Adaptive Loss Balancing (GradNorm)^70^. According to this approach, loss weights have to be dynamic, meaning that a specific task weight differs given different inputs, compared to GradNorm where the task weight is static as it is identical among all batches.

DLW assumes that the task-specific loss is informative for balancing different tasks. For each task and batch, DLW considers the loss ratio between the current loss and the initial loss (Algorithm B1 in Appendix B and Eq. 3), which is a proxy for how well the model has trained. Poorly trained tasks have ratios close to one and contribute more to the overall loss and gradient. Having applied the DLW approach, the loss for the proposed disease segmentation model, based on MTL, is calculated as follows:

3 $$Loss= {w}_{B}^{cls}\times {L}_{B}^{cls}+ {w}_{B}^{seg}\times {L}_{B}^{seg} ={\bigg(\frac{{L}_{B}^{cls}}{{L}_{(0,i)}^{cls}} \bigg)}^{\alpha }\times {L}_{B}^{cls}+ {\bigg(\frac{{L}_{B}^{seg}}{{L}_{(0,i)}^{seg}}\bigg)}^{\alpha }\times {L}_{B}^{seg}$$

where ${w}_{B}^{cls}$ and ${w}_{B}^{seg}$ are dynamic weights for the classification and segmentation tasks, ${L}_{B}^{cls}$ and ${L}_{B}^{seg}$ are the losses obtained on batch $B$, ${L}_{(0,i)}^{cls}$ and ${L}_{(0,i)}^{seg}$ are the first batch losses, $\alpha$ is a hyperparameter balancing the influence of the task-specific weights and equal to 0.5 for the proposed MTL model. As $\alpha$ goes to 0, DLW approaches standard MTL.

### Post-processing: severity scoring and visualization

**Figure 6:**
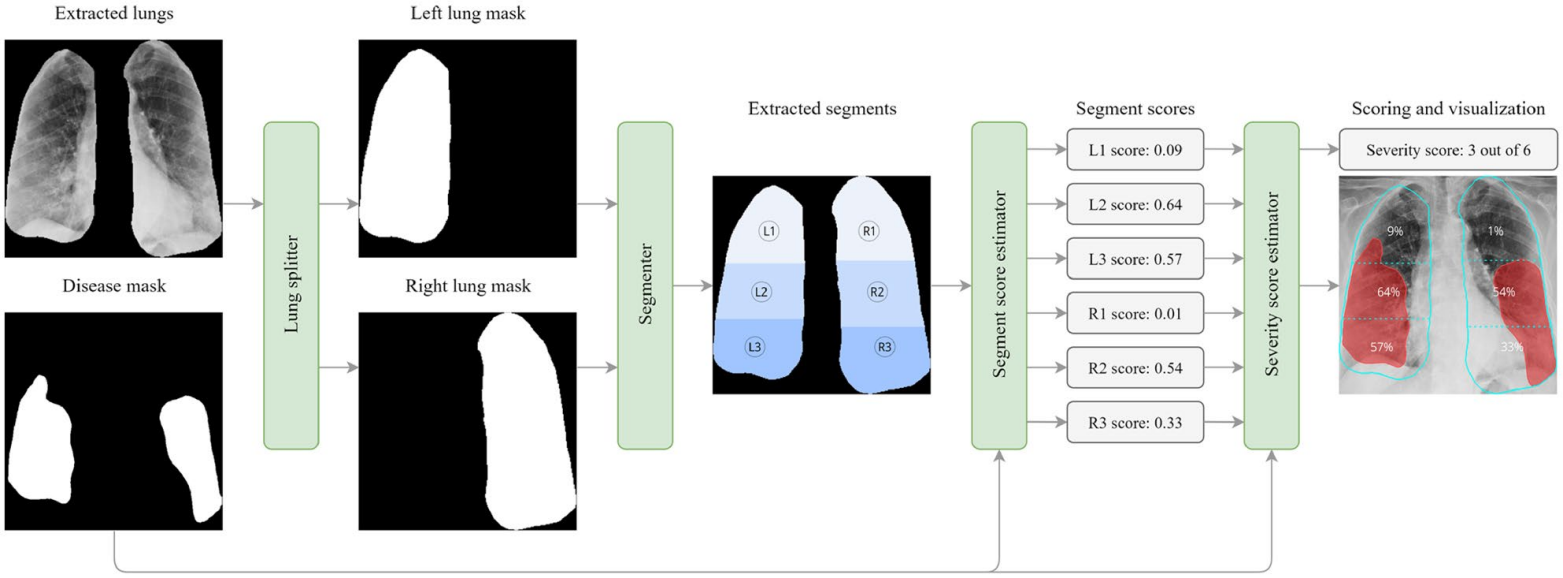
Post-processing block for scoring estimation.

For quantification, qualification, and visualization of the affected area of the lungs, we utilize the post-processing block, as shown in Fig. 6, which is primarily used to estimate the scores for lung segments as well as the overall score. Having obtained the extracted lungs from Stage I, first, we separate the two lungs. Here, we employ an algorithmic application of graph theory called Block-Based Connected Components^71^ which is used to determine the connectivity of BLOB-like regions in a binary image. Once this algorithm is applied, we detect the two biggest BLOBs which represent the lungs in an image. After the lungs are detected we take each BLOB, representing a single lung mask, and nullify other pixels using the bitwise AND operator. Having performed this set of operations, the lung splitter outputs two separated lung masks ${M}_{l}$ (mask of left lung) and ${M}_{r}$ (mask of right lung).

Once two binary lung masks are obtained, we use a segmenter block to divide each lung into three segments. Although similar to clinical practice, the frontal X-ray image is divided into three zones per lung (total of 6 zones)^72^: upper, middle, and lower zone. The upper zone extends from the apices to the superior portion of the hilum. The middle zone spans the space between the superior and inferior hilar margins. The lower zone extends from the inferior hilar margins to the costophrenic sulci. This approach, however, does not consider the area of the estimated zones that lead to non-uniform segment division and, in turn, lead to a different impact made by each segment on the estimation of the total score. Furthermore, such a methodology is dependent on the image alignment, leading to some solutions, for instance, BS-Net^30^, integrating an alignment block inside their workflow. Besides being of high complexity, due to the application of a neural network, the alignment block is typically placed right before the core model, as such any error originating from this block may significantly influence the error of the entire pipeline. In this regard, we propose a segmenter that is invariant to affine and geometric transformations, and divides each lung into three segments, maintaining a consistent area within each segment and across both lungs (Algorithm B2 in Appendix B).

To divide each lung into three equal-sized segments, we apply a binary search algorithm, which runs twice, to compute both the upper ${y}_{top}$ and lower ${y}_{bot}$ coordinates. Initially, the segmenter searches for the upper coordinate ${y}_{top}$ in the interval $[0, height]$, so that the sum of pixel values of mask $M$, in the range $[0, {y}_{top}]$, is as close to $\frac{1}{3}\times S$ as possible, where $S$ is the total sum of the mask pixel values equal to ${\sum }_{i=1}^{width}{\sum }_{j=1}^{height}{M}_{ij}$. Finally, this procedure is repeated and the segmenter searches for the lower coordinate ${y}_{bot}$ in the interval $[0, height]$, so that the sum of pixel values of mask $M$, in the range $[0, {y}_{bot}]$, is equal to $\frac{2}{3}\times S$. Having performed this procedure, the segmenter outputs two $y$ coordinates (upper and lower) per each lung (total of 4 coordinates i.e. ${y}_{left}^{top}$, ${y}_{left}^{bot}$, ${y}_{right}^{top}$, and ${y}_{right}^{bot}$).

Having estimated the lung mask, disease mask, and the limits of the six lung segments, we utilize two estimators (Algorithm B3 in Appendix B) for the computation of the severity score per segment, and the total severity score for a given subject. For this procedure, we take the intersection of the predicted mask of the disease and the segments for each lung obtained by the segmenter. If the intersection of these regions is big enough, meaning it is more than a predefined threshold value $T$, we count this part as 1, otherwise 0. At the end of the proposed pipeline, the severity score estimator sums up all values for each segment and gives the total score, which falls in the range of 0 to 6.

### Hyperparameter tuning

In the proposed pipeline, both stages rely on neural networks. Moreover, Stage II is based on an MTL neural network, requiring hyperparameter tuning to obtain optimal performance. Despite different techniques being explored in addressing the problem of neural network hyperparameter optimization, such as Neural Architecture Search^73^ by Google and Generative Synthesis^74^ by DarwinAI, one challenge stands out—computational cost. Even though Grid Search and Random Search^75^ are more efficient in terms of time spent for the optimization, they are completely uninformed by past trials, and, as a result, often spend a significant amount of time evaluating irrelevant hyperparameters. Thus, the Bayesian methodology^76^ was chosen as the main optimization technique and the hyperparameter optimization task comes down to the following minimization task:

4 $${x}^{*}=\mathrm{arg}\underset{x\in X}{\mathrm{min}}{f}^{cls}(x)+\mathrm{arg}\underset{x\in X}{\mathrm{min}}{f}^{seg}(x)$$

where ${f}^{cls}(x)$ and ${f}^{seg}(x)$ represent classification and segmentation objective scores to be minimized and evaluated on the validation subset, ${x}^{*}$ is the set of hyperparameters that yields the lowest value of the overall score, and $x$ can take on any value in the domain $X$. We note that due to the MTL architecture ${f}^{cls}(x)$ is only used for networks in Stage II, otherwise, it is equal to 0.

In essence, Bayesian optimization is a probability model and its key advantage is compatibility with a black-box function, whilst being data-efficient and robust against noise. However, it works poorly with parallel resources as the optimization process is sequential. In this regard, we used an additional extension of the Successive Halving algorithm^77^ called HyperBand^78^. In contrast to HyperBand, Successive Halving suffers from a trade-off between the selection of the number of configurations and the number of cuts while allocating the budget. As a solution, HyperBand proposes to perform the Successive Halving algorithm with different budgets to find the best configurations. HyperBand evaluates whether tuning has to be stopped or permitted to continue at one or more pre-set iteration counts, called brackets. When a trial reaches a bracket, its metric value is compared to previously reported metric values and the trial is terminated if its value is too high (minimization goal) or too low (maximization goal). The goal of the tuning we perform is related to the maximization of a segmentation score, the Dice similarity coefficient (DSC). In order to specify the bracket schedule and maximize the aforementioned metric, we use:

- ${I}_{max}=16$ (the maximum number of iterations);
- $S=2$ (the total number of brackets);
- $ETA=2$ (the bracket multiplier schedule).

The brackets are computed using the equation ${B}_{k}={I}_{max}/{ETA}^{k}$, where $k$ is the bracket number. The latter means that the brackets for tuning are $[16/{2}^{1}, 16/{2}^{2}]$ equaling $[8, 4]$. In addition to Hyperband, we use a complementary stopping strategy, the Early Stopping algorithm, that helps to reduce the computation time. This algorithm is used as a regularization, which allows for the removal of poorly performing trials and attempts at more configurations. The key settings of the Early Stopping algorithm used for tuning are:

- Metric to be monitored: Dice similarity coefficient;
- ${\Delta }_{min}=0.01$ (minimum change in the monitored quantity to qualify as an improvement);
- $e=6$ (number of epochs with no improvement after which the training is stopped).

In connection, instead of a blind repetition algorithm on top of Successive Halving, we use a Bayesian optimization with constraints imposed by two algorithms: HyperBand and Early Stopping. In such an approach, Early Stopping acts as an intra-regularizer that estimates the performance of a single trial in an epoch-by-epoch manner. Whereas, HyperBand plays the role of an extra-regularizer which estimates the performance between trials and terminates poorly performing ones in a bracket-by-bracket manner.

Conducting a hyperparameter search is a non-trivial task due to the variability in hyperparameter priority when it comes to tuning them i.e. models are more sensitive to certain hyperparameters than others, necessitating a more impactful strategy^79^. As a result, we did not optimize hyperparameters such as batch size, non-linearity type, optimizer options, kernel sizes, etc. However, we pay attention to the encoder architecture, input image size, loss function, optimizer, and the learning rate. In Table C1 of Appendix C, we summarize the explored hyperparameters along with their values used during tuning.

The dataset used during tuning differs from the one we use in training. First, the testing subsets are not used for tuning purposes and the termination or early stopping of the trials are based on the DSC value computed on a validation subset. Second, the overall tuning dataset includes fewer images than the dataset used for final training and testing. A comparison of the datasets used in both steps is reflected in Table 4. According to the displayed distribution, we use 10% of the whole dataset for the tuning of lung segmentation networks and 50% for the tuning of disease segmentation networks. Such a difference is explained by the fact that the region of both lungs is more distinctive than the COVID-19 affected regions. The typical appearance of such regions presents ground-glass opacities (with or without consolidation) or a “crazy-paving” pattern, which is the appearance of ground-glass opacities with superimposed interlobular septal thickening and intralobular septal thickening. Such patterns present in images forced us to increase the tuning dataset in order to find the best configuration of the studied networks.

**Table 4:** Comparison of the datasets used in both stages.

Stage	Phase	Subset			Total
		Training	Validation	Testing	
Stage I (lung segmentation)	Tuning	544	137	–	681/10%
	Training	5446	682	682	6810/100%
Stage II (disease segmentation)	Tuning	521	134	–	655/50%
	Training	1089	136	139	1364/100%

### Hyperparameter correlation and importance

In addition to the best configurations, we estimate which of the investigated hyperparameters are the best predictors and are highly correlated with the desirable metric, the DSC. For hyperparameter quantification, we compute two metrics: correlation, between the hyperparameter and the chosen metric, and importance. Correlation ranges from − 1 to 1, where positive values represent a positive linear correlation, negative values represent a negative linear correlation, and a value of 0 represents no correlation. Generally, a value greater than 0.7 in either direction represents a strong correlation. Correlation, alone, cannot capture second-order interactions between inputs and it can get messy when comparing inputs with different ranges. As such, we estimate a complementary metric, importance, where we train a random forest with the hyperparameters as inputs and the metric as the target output, and report the feature importance values for the random forest. We were inspired by a methodology proposed by Jeremy Howard, who has pioneered the use of random forest feature importance to explore hyperparameter spaces at Fast.ai^80^. This is in contrast to the adoption of linear regression for the task at hand, which works well if the dataset is properly prepared, as random forests are more tolerant of different data types and scales. We note that hyperparameters with importance values of 0.05 and lower are likely not important.

Below we outline key factors for the interpretation of correlation and distinguishing it from the importance:

- Correlation shows evidence of association, but not necessarily causation;
- Correlations are sensitive to outliers which may turn a strong relationship into a moderate one, especially if the investigated sample size of hyperparameters is small;
- Correlations only capture linear relationships between hyperparameters and metrics i.e. if there is a strong non-linear relationship, it may not be captured.

Disparities between importance and correlation result from the fact that importance accounts for interactions between hyperparameters, whereas correlation only measures the effect individual hyperparameters have on metric values. Secondly, correlations strictly capture linear relationships, whereas importances can capture more complex ones. Nevertheless, both importance and correlation are powerful metrics for the understanding of how hyperparameters influence model performance, and are both used in our study.

### Model training

Once the tuning of networks in both stages is performed, we train nine models with their best configurations i.e. the best configuration per model (U-net, U-net++, DeepLabV3, etc.). During training, we used the Early Stopping strategy, similar to that described in “Hyperparameter tuning”. Additionally, we employ a set of augmentation transformations that are used during both the tuning and training steps. Besides allowing us to increase the size of the dataset, augmentation acts as a regularizer and helps reduce overfitting during model training. The proposed augmentation workflow consists of the following transformations:

- Contrast limited adaptive histogram equalization with a probability of 20%;
- Random-sized crop with a probability of 20% (weight-to-height ratio of the crop equal to 1, the range of crop is picked from $0.7\times {I}_{h}$ to $0.9\times {I}_{h}$, where ${I}_{h}$ is the source image height);
- Rotation with a probability of 50% (a random angle is picked from –15° to + 15°);
- Horizontal flip with a probability of 50%;
- Random brightness and contrast adjustment with a probability of 20% (factor range for changing both brightness and contrast is picked from − 0.2 to + 0.2).

In contrast to the tuning step, where the batch size was chosen as a fixed value equal to 4, the training step does not use a fixed batch size. Since the studied models are of different complexity, they require different memories for training, for a fixed batch size. In this regard, we decided to equalize the trained models by the GPU memory utilization i.e. each model is trained using a batch size allocating approximately 90–100% of GPU memory.

## Results

### Hyperparameter tuning

Following the approach described in “Hyperparameter tuning”, we sequentially tuned the networks for both stages, lung and disease segmentation. We found that approximately 100 runs turn out to be minimally enough to select optimal hyperparameters for the lung segmentation networks. However, to extend the hyperparameter space, we tripled the number of runs. Each network was trained with a batch size of 4 on NVIDIA GeForce RTX 3090 24 Gb. A small batch size was selected due to the physical limitation of the GPU memory and the out-of-memory error thrown during training. We decided to bias our focus to the encoder rather than the batch size. In this regard, we estimated a wide variety of encoders and models rather than several lightweight/middleweight models and encoders with different batch sizes. Besides the accuracy metric (DSC), we estimated the number of parameters for each model and its complexity. The complexity is represented by the theoretical amount of multiply-accumulate (MAC) operations in CNNs.

Having performed 300 trials with different hyperparameter combinations, we found all possible optimal solutions for lung segmentation (Table 5). According to our results, the Adam optimizer, or its variants, turned out to be the optimal solution for network training and effective convergence. The optimal learning rate falls into the range of 10^–3^ to 10^–4^, while the median input size for the training and inference of an optimal network is equal to 512 × 512 pixels. In terms of segmentation accuracy i.e. DSC, the best-performing models are FPN (0.949), DeepLabV3 + (0.948), and Linknet (0.946). While models with the lowest complexity (MAC) are PAN (0.3 G), DeepLabV3 + (2.2 G), and FPN (9.1 G). Having compared all nine models, DeepLabV3 + turned out to be the optimal network in terms of the accuracy-complexity ratio i.e. DSC-MAC ratio.

**Table 5:**
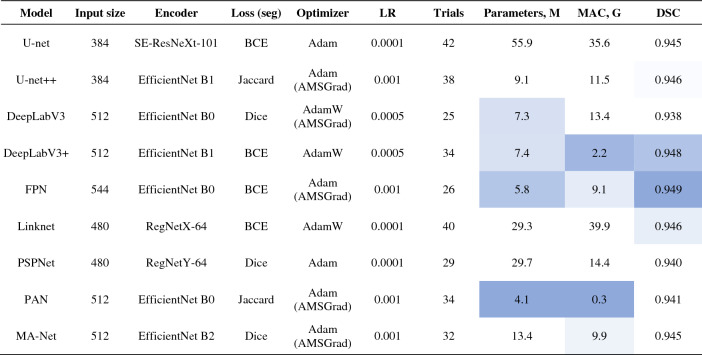
Best configurations for lung segmentation networks.

**Figure 7:**
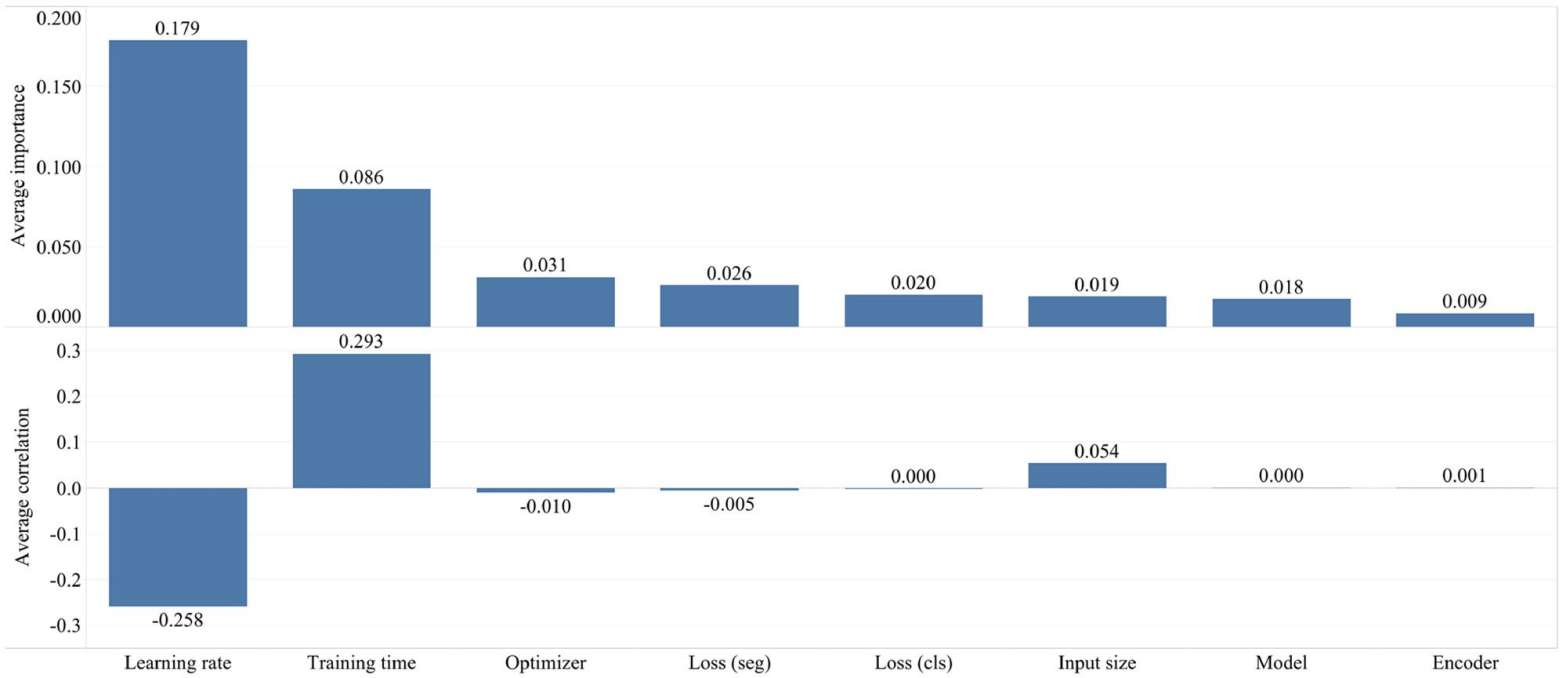
Average hyperparameter importance and correlation for lung segmentation.

As we described in “Hyperparameter correlation and importance”, we additionally estimate two metrics (correlation and importance), showing the degree to which each hyperparameter was useful in predicting the chosen metric. Below, in Fig. 7, we summarize the average correlation and importance values. As we described before, we calculate the importances using a tree-based model rather than a linear model as the former is more tolerant of both categorical data and data that is not normalized. Based on our results, the learning rate and the training time turned out to be the most important hyperparameters, significantly affecting the accuracy of the studied networks. Also, these metrics correlate significantly with the estimated segmentation metric (Dice similarity coefficient). However, they are of opposite correlation nature with the desired metric i.e. higher DSC values with smaller learning rates. Furthermore, the training time and DSC are positively correlated, meaning networks that are trained longer result in a better accuracy performance. Additionally, in Fig. C1 of Appendix C, we provide the original correlation and importance values per hyperparameter.

To find the optimal configurations for COVID-19 segmentation networks, we employ a similar strategy with the key difference being the presence of a regularization branch. The optimization of these networks refers to the hyperparameter tuning of an MTL model. Another difference is connected with the number of runs we estimated. In contrast to lung segmentation networks, the number of runs for the hyperparameter tuning was increased by 7 times resulting in 2,100 runs. Such a big difference between the two segmentation approaches is related to the deeper exploration of the hyperparameter space of the disease segmentation networks because (a) the latter plays a crucial role in the proposed scoring workflow and (b) the segmentation of indistinguishable COVID-19-affected regions is of higher complexity than the segmentation of the more distinctive lungs. Having performed the hyperparameter tuning of the COVID-19 segmentation networks, we obtained the results shown in Table 6. Similarly, the Adam optimizer and its variants proved to be optimal. The most accurate models are U-net (0.894), PSPNet (0.879), and MA-Net (0.876). PSPNet with the EfficientNet B0 encoder has the lowest complexity (0.1 G) which leads to the best performance as compared to other solutions. The second and third lightweight models are Linknet (0.5 G) and FPN (4.5 G).

**Table 6:**
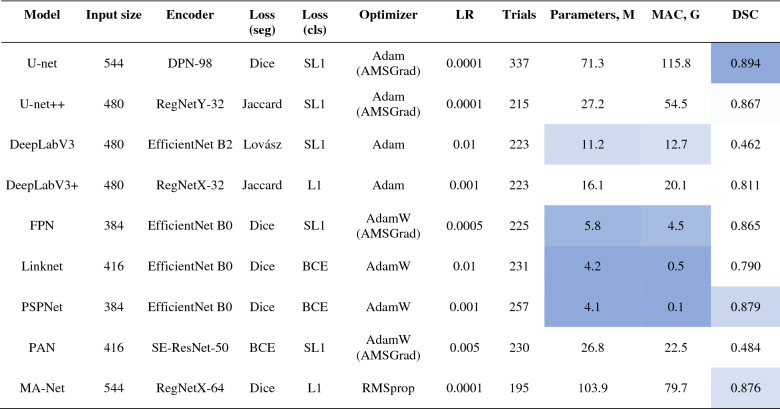
Best configurations for COVID-19 segmentation networks.

**Figure 8:**
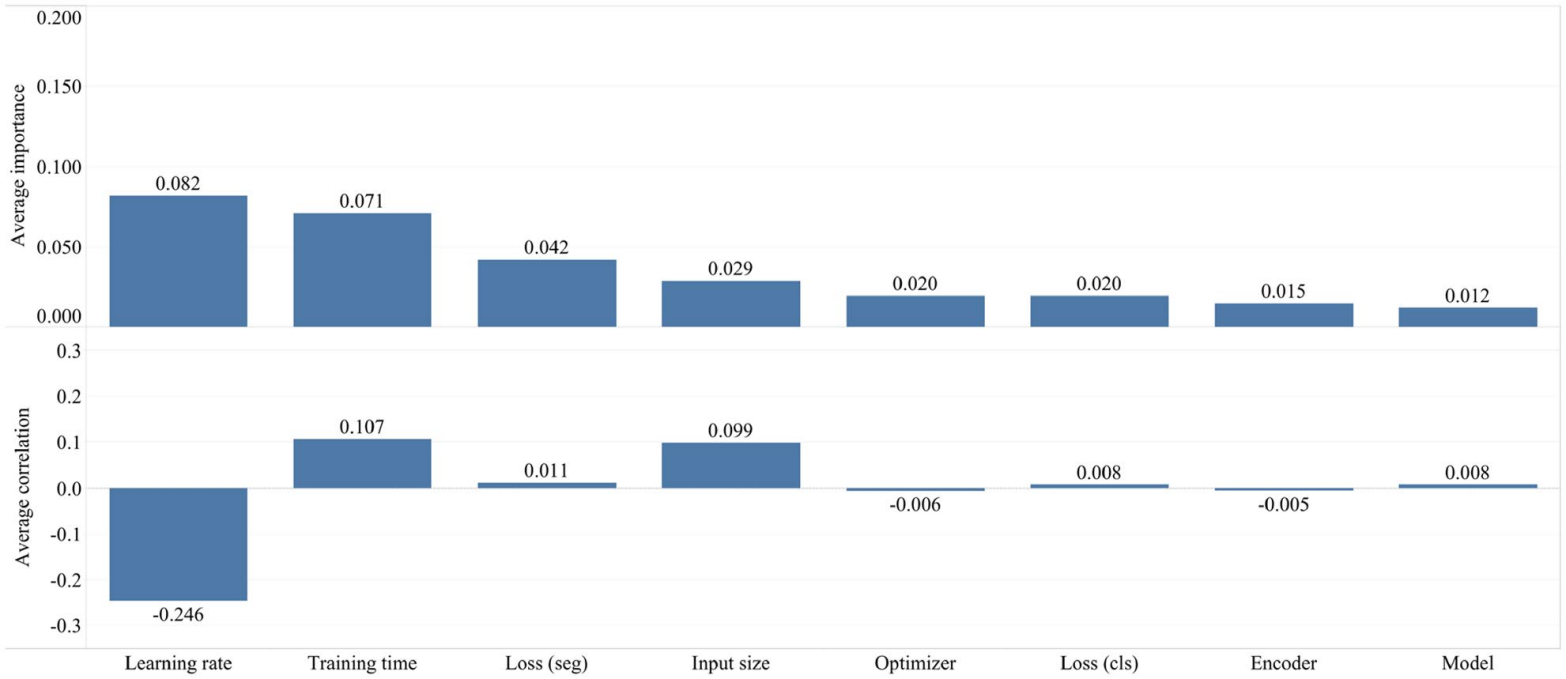
Average hyperparameter importance and correlation for COVID-19 segmentation.

Figure 8 displays the average correlation and importance per hyperparameter for the COVID-19 segmentation networks. We observe that the learning rate and the training time are the most important hyperparameters. Additionally, in Fig. C1 of Appendix C, we provide the original correlation and importance values per hyperparameter.

### Model training

Having performed hyperparameter optimization and found the best configurations, we trained nine lung segmentation networks according to the methodology described in “Model training”. Once the networks were trained and validated, we tested them, summarizing their main specifications and the Dice similarity coefficient in Table 7. Additional metrics related to the quality of lung segmentation are reflected in Appendix D, Table D1. As can be seen, all networks have a high level of segmentation accuracy, however, some of them are computationally expensive (U-net, Linknet, and PSPNet) with relatively similar values of DSC. In this regard, DeepLabV3 + (DSC_test_ = 0.963, parameters = 7.4 M, MACs = 2.2 G) is chosen as an optimal solution which is used in our scoring pipeline as the core block for Stage I**.**

**Table 7:**
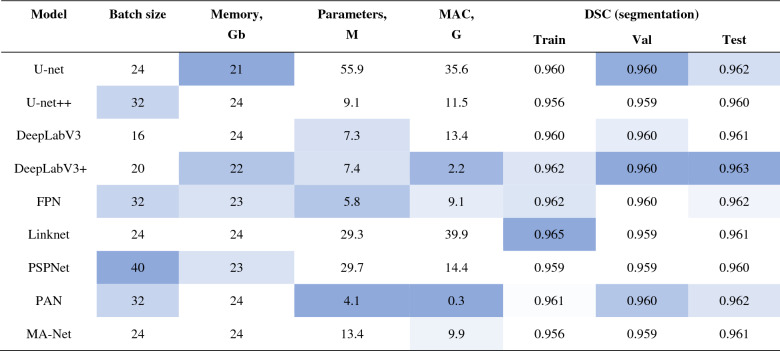
Results of the fully trained lung segmentation networks.

Similarly, we trained nine MTL networks for both segmentation and classification of COVID-19 and normal cases, where the latter is connected to patients with no diseases or no findings. In Table 8, we give a summary of the obtained results. Since the networks of Stage II are MTL-based, we report both segmentation and classification accuracies (DSC and F1 score). More detailed results, including metrics such as Accuracy, Precision, Recall, Dice similarity coefficient, and F1 score, are presented in Appendix D, where Table D2 is devoted to the segmentation results, while Table D3 to the classification results. As can be seen, U-net and FPN proved to be the most accurate networks in both classification and segmentation tasks. U-net achieved an F1 score, on the testing subset, of 0.985, which is 1.9% (0.019) higher than the F1 score of FPN which is 0.966. However, in terms of the number of parameters, U-net is almost 26 times more computationally expensive than FPN (115.8 M vs 4.5 M), which usually leads to a lower prediction speed. In this regard, both networks may be employed as the optimal solution for Stage II, depending on the required performance (processing time) and accuracy (DSC and F1).

**Table 8:**
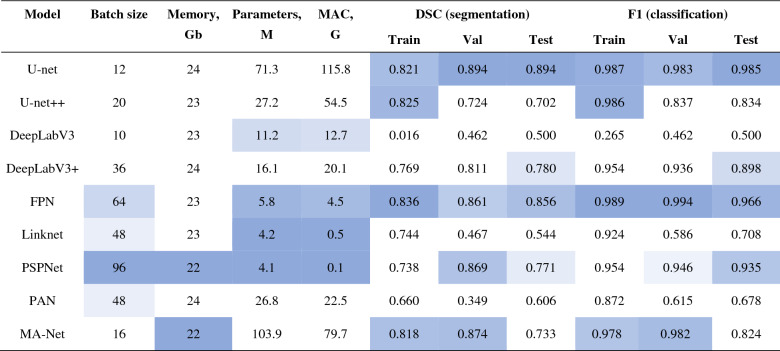
Results of the fully trained COVID-19 segmentation networks.

### Scoring results and comparison with the state-of-the-art solutions

After training the networks with their optimal configurations, we obtained nine networks for each stage (18 networks in total). As discussed (“Model training”), all networks perform lung segmentation with approximately the same accuracy. However, several networks outperform others in terms of computational complexity and prediction time. To this extent, for testing the proposed scoring pipeline, we choose DeepLabV3 + as the core network of Stage I. On the other hand, we do not freeze Stage II with the usage of only one network. We test all nine COVID-19 segmentation networks which are followed by DeepLabV3 + .

In “Post-processing: severity scoring and visualization” and in Algorithm B3 of Appendix B we introduced a threshold parameter $T$. In order to estimate a score of each lung segment, we take the intersection of the predicted disease mask and the corresponding lung segment. If the intersection of these two regions is greater than or equal to a predefined threshold $T$, we count this segment as 1 (affected by the disease), otherwise 0 (non-affected or slightly affected by the disease). At the end of the proposed pipeline, the severity score estimator sums up the values for each segment and gives the total score which falls in the range of 0 to 6. Based on the training and validation subsets, before testing our workflow, we chose an optimal threshold $T$ (Table 9) which is different for each network in Stage II. We estimate threshold values based on the lowest MAE and RMSE values. The optimal threshold $T$ is chosen based on the minimum MAE for the following workflow testing.

**Table 9:** Optimal thresholds estimated for severity scoring.

Stage I model	Stage II model	MAE		RMSE	
		Min	Threshold	Min	Threshold
DeepLabV3 +	U-net	0.28	77	0.61	77
	U-net + +	0.41	84	0.90	99
	DeepLabV3	1.58	74	2.75	69
	DeepLabV3 +	0.37	77	0.80	77
	FPN	0.33	66	0.69	66
	Linknet	0.38	38	0.81	41
	PSPNet	0.42	97	0.88	97
	PAN	0.52	102	1.05	102
	MA-Net	0.32	79	0.70	79

To strictly validate the proposed workflow, we test it on an unseen dataset (testing subset) which is described in “Stage II: disease segmentation and scoring dataset”. Additionally, we estimate the workflow performance on the overall testing subset, including both COVID-19 and normal cases (Table 10), on the testing subset with only COVID-19 cases (Table E1 in Appendix E), and on the testing subset with only normal cases (Table E2 in Appendix E). Besides the internal comparison of different models within Stage II, we compare the obtained results against tailor-made state-of-the-art solutions used for disease scoring, namely BS-net and COVID-Net-S. Although MAE and RMSE of BS-net and COVID-Net-S on the pure COVID-19 dataset are of relatively acceptable level, the error on the dataset with healthy subjects (normal cases) turned out to be high. Meaning that most healthy subjects are usually scored as mild or intermediate COVID-19 cases. To compare the obtained scoring results and make them visually distinct, we use a blue-white-red color scale, where blue refers to a better performance, red refers to a worse performance, and white is intermediate.

**Table 10:**
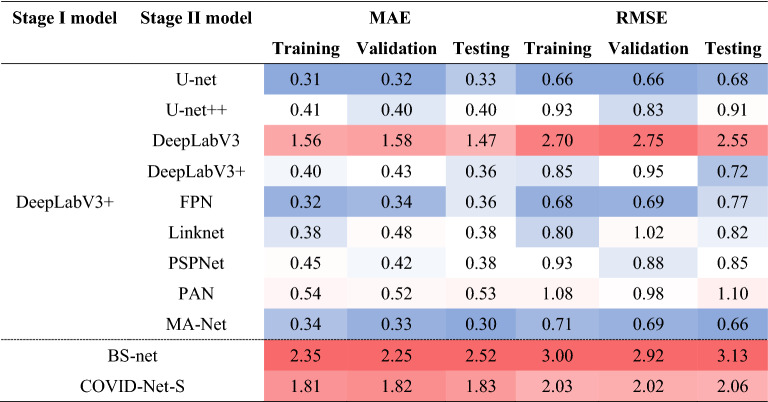
Scoring performance estimated on a dataset of COVID-19 and normal cases.

From our studies, we found several optimal solutions for lung disease scoring. The most accurate variants for the proposed workflow are based on U-net, FPN, or MA-net, used for disease segmentation in Stage II. The MA-Net-based workflow achieved the lowest MAE of 0.30 on the testing subset, while U-net and FPN MAEs are of similar levels and equal to 0.33 and 0.36 respectively. It should be noted that we consider MAE and RMSE dynamics over the training, validation, and testing subsets to choose the best three networks. As shown in Table 10, the obtained optimal solutions did not overfit. We note that the key to the comparison of the proposed workflow with state-of-the-art solutions is the estimation of both COVID-19 cases (Table E1 in Appendix E) and normal cases (Table E2 in Appendix E) separately.

**Figure 9:**
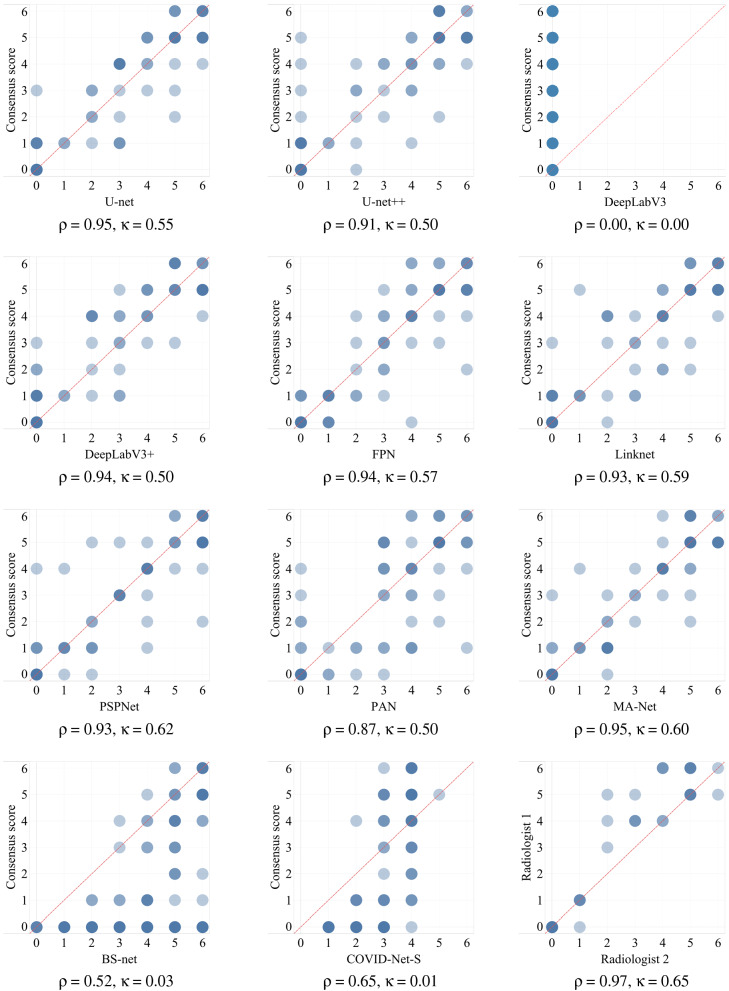
Relationship between the consensus score and the score of different networks.

While MAE and RMSE provide formal quantifications of the overall performance of each network and allow for a formal comparison between the networks, they do not evaluate the performance of networks based on the underlying score values. The latter is desired since some networks can perform better on higher scores, some on lower scores, and some can be the same for all score values. To address this, visual summaries of each network’s performance are provided for the testing subset as a heatmap in Fig. 9, where a darker color indicates a higher density of points and those points on a red dashed line indicate perfect agreement between the network and the consensus score. The comparison between radiologists scoring is also provided in Fig. 9 (bottom-right chart). The network’s score and the consensus score on the red line indicate perfect agreement while deviations in each side reflect whether the network underscores or overscores. A darker color on these plots represents the density of points for score combinations i.e. the deeper the color the more outcomes are within the given combination of scores. For formal numeric comparisons, the correlation coefficient (ρ) and Cohen’s kappa (κ) were computed for each network. MA-Net, U-net, and DeepLabV3 + had the largest correlation values (ρ) equal to 0.95, 0.95, and 0.94 respectively. At the same time, the largest values of Cohen’s kappa (κ) were for PSPNet (0.62), MA-Net (0.60), and Linknet (0.59). The formal numeric comparison between the radiologists on the testing subset had the correlation values (ρ) equal to 0.97 and Cohen’s kappa (κ) equal to 0.65.

**Figure 10:**
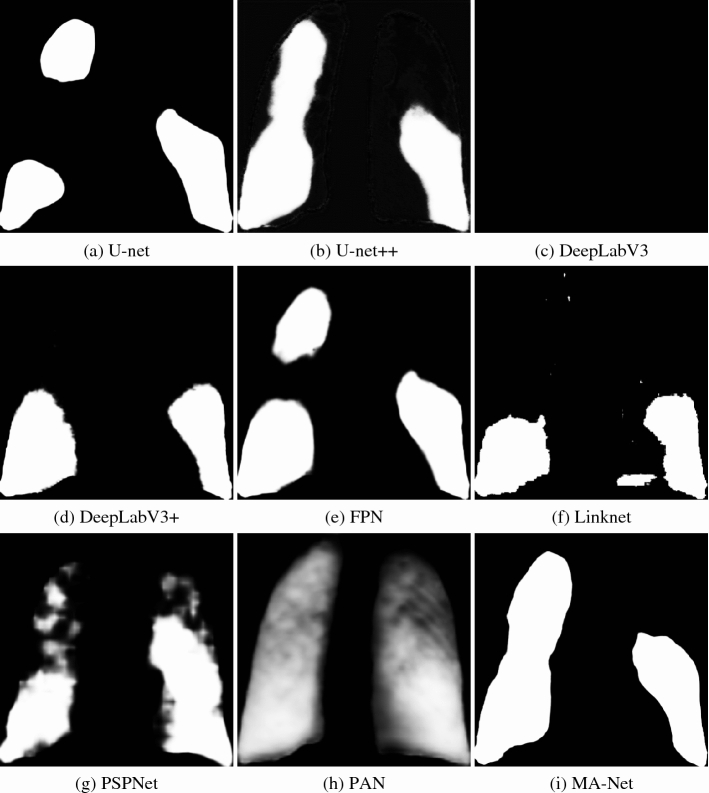
Comparison of the probability maps of a COVID-19 subject from the ACCD dataset.

**Figure 11:**
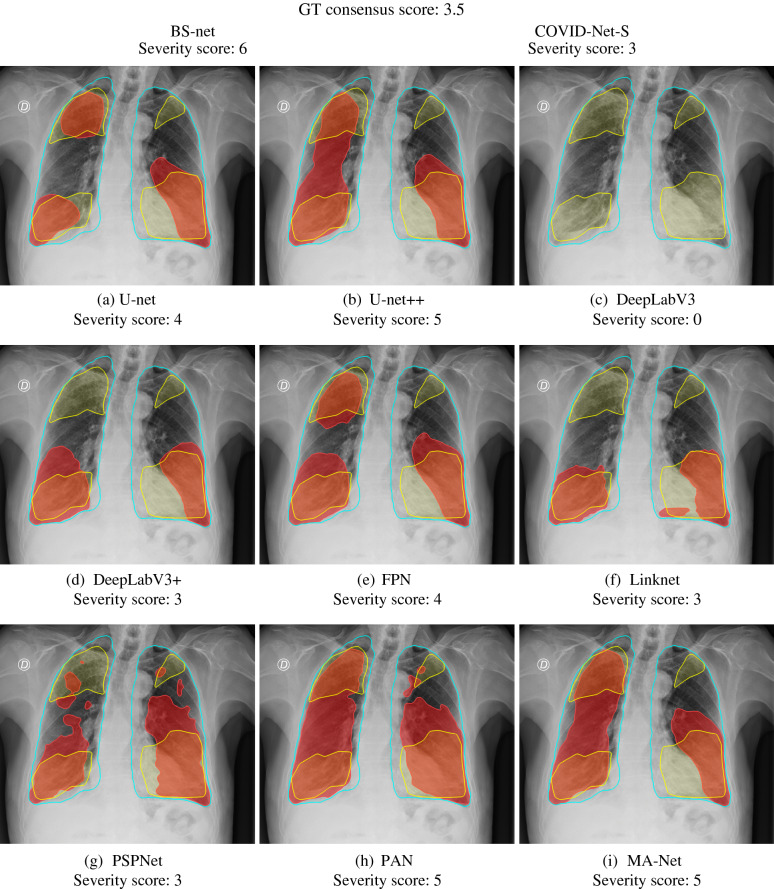
Comparison of the segmentation and severity score estimation of a COVID-19 subject from the ACCD dataset. A cyan delineation refers to the lung segmentation obtained by Stage I; a red mask is a disease mask obtained by Stage II; a yellow mask refers to the ground-truth segmentation of the disease.

For visualization purposes, we provide probability maps of each network in Fig. 10 and Appendix F. Each probability map represents an output of Stage II scaled on a 0–1 range. As shown, all probability maps are of different nature. For instance, some networks such as U-net +  + , PSPNet, and PAN showcase blurring mask edges. Linknet usually outputs many erroneous binary objects, while DeepLabV3 cannot segment the COVID-19-affected region. In turn, U-net, FPN, and MA-Net perform well, segmenting with no artifacts or blurred borders. The latter confirms that these three networks achieved the best results in severity scoring (Table 10, Tables E1 and E2 in Appendix E). Figure 11 showcases CXR images of a subject taken from the ACCD dataset, where we additionally provide the ground-truth severity score and the scores obtained by BS-net and COVID-Net-S. Additional cases taken from other COVID-19 datasets (CRD, CCXD, and FCXD) are reflected in Appendix G.

**Figure 12:**
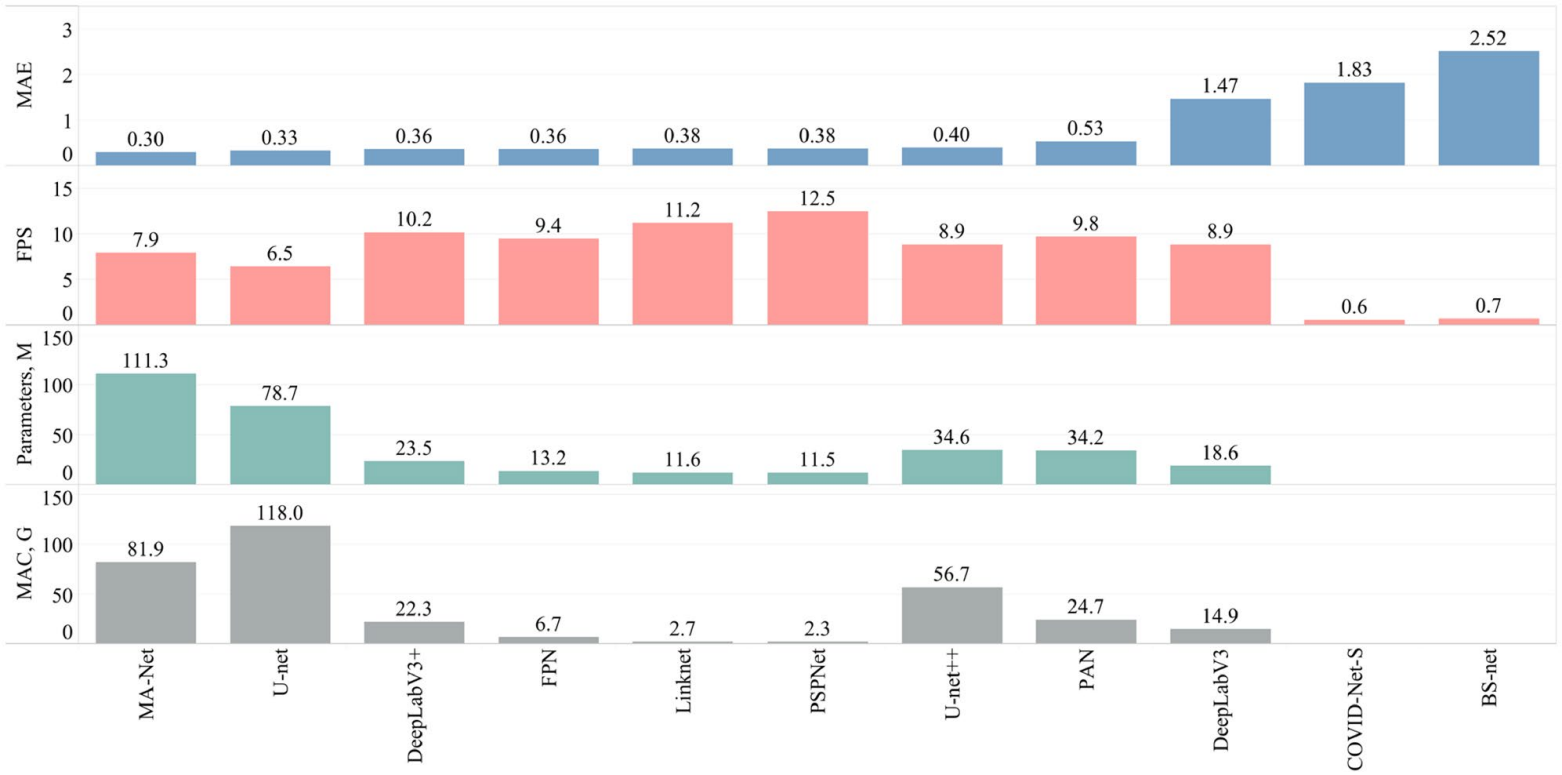
Overall comparison of the obtained solutions.

For the overall comparison of the proposed solutions, we showcase MAE estimated on the testing subset, the frame rate (FPS), the number of overall parameters, and MAC in Fig. 12. The Y-axes “Parameters” and “MAC” refer to the overall number of parameters and the theoretical amount of multiply-accumulate operations for both stages of the proposed workflow. Similar to the accuracy estimation, we choose DeepLabV3 + as the core network of Stage I. In Stage II we tested nine networks. All networks were tested in the evaluation mode meaning that (a) normalization or dropout layers work in evaluation mode instead of training; (b) the automatic differentiation engine is deactivated. Adoption of the evaluation mode reduces memory usage and speeds up computations turning the back-propagation over the network. The main GPU used for testing is NVIDIA RTX 2080 Ti 11 Gb. The best performance (12.5 images/s) resulted in a proposed pipeline consisting of DeepLabV3 + (Stage I) and PSPNet (Stage II) whilst ranking sixth by MAE of the severity score. The most accurate solution consisted of DeepLabV3 + (Stage I) and MA-Net (Stage II), ranking eighth in the level of performance (7.9 images/s). On the other hand, the prediction speed of the tailor-made solutions, BS-net and COVID-Net-S, turned out to be the lowest making up 0.7 and 0.6 images/s respectively.

## Discussion

### Issue of the source data

In this study, we utilize four publicly available COVID-19 datasets. According to the available protected health information, these datasets represent patients from over 40 countries, 107 cities, and 40 organizations. However, from a scientific and statistical point of view, we cannot ensure that all of the specified and/or unspecified medical organizations follow the same detailed protocol while data gathering. According to the generally accepted data collection rules^81,82^, the study design should be reproducible, so that the protocol can be followed by any other research party. All of the data needs to be gathered in a consistent manner and if data is collected by different individuals, it must be guaranteed that there is a sufficient degree of inter-rater reliability. In this regard, we cannot verify that there was no collection and processing biases in the data used for the analysis.

### Comparison with existing scoring solutions

Having estimated both state-of-the-art solutions (BS-net and COVID-Net-S), we found them not suitable for the usage on CXRs of healthy patients, signifying the limits of their usage in daily clinical practice. As we described in “Scoring results and comparison with the state-of-the-art solutions”, BS-net and COVID-Net-S fail on the normal CXR cases and their MAEs (on a scale from 0 to 6) equate to 3.25 and 2.20 respectively. Such an error rate skews the networks to score healthy patients as mild or intermediate COVID-19 cases. However, even if we focus solely on COVID-19, the proposed workflow outperforms both BS-net and COVID-Net-S in disease scoring. Our solution based on DeepLabV3 + for lung segmentation and MA-Net for disease segmentation achieved an MAE of 0.69 (Table E1 in Appendix E), while MAEs of BS-net and COVID-Net-S are equal to 1.45 and 1.29 respectively.

From an architectural perspective, both BS-Net and COVID-Net-S are relatively lightweight. COVID-Net-S utilizes a lightweight residual projection-expansion-projection-extension design pattern discovered by the machine-driven design exploration strategy and uses 1 × 1 convolution layers and 3 × 3 depth-wise convolution layers. The authors of BS-net also choose a lightweight solution for the processing of input images which is based on ResNet-18, while the segmentation is performed by a nested version of U-net, U-net++. We are inclined to believe that such lightweight networks do not generalize well for COVID-19 and pneumonia cases and that, in turn, leads to low-quality scoring, requiring additional validation by radiologists or clinicians. In contrast, the proposed workflow is based on network architectures with proven stability and generalization ability on a wide variety of tasks, including medical cases. Moreover, having hyper-tuned the networks in both stages of the workflow, we found optimal solutions in both accuracy and performance. In contrast to BS-net and COVID-Net-S, the proposed workflow, based on modern architectural solutions, outperforms them in prediction speed (Fig. 12). This means that despite the lightweight design of both BS-net and COVID-Net-S, these networks are of high complexity and potentially include more parameters than that of the modern networks highlighted in this study.

**Figure 13:**
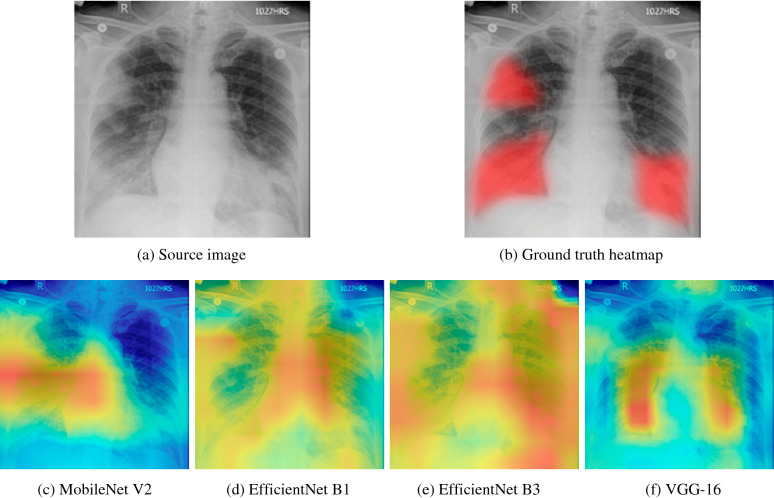
Grad-CAM visualization of classification network heatmaps for a COVID-19 finding.

The majority of the current disease classification solutions focus, primarily, on distinguishing whether an infection is present or not, without paying much attention to where the network is looking. Previously^13^, we extended the classification of COVID-19 and pneumonia by utilizing a popular visualization technique known as Grad-CAM^83^. Using Grad-CAM, we validated where the four best-performing networks (MobileNet V2, EfficientNet B1, EfficientNet B3, VGG-16) were focusing, verifying that they are properly looking at the correct patterns in the image and activating around those patterns. We noticed that some networks were not focusing on image patterns of interest, instead, activating around patterns that lead to incorrect predictions (see Fig. 13c–e). Moreover, complex patterns of COVID-19 and pneumonia distinguished solely by radiologists forced us to develop the proposed method, where the considered models focus their attention explicitly on lung areas with additional regularization and removal of unnecessary regions in the decision making.

The Grad-CAM technique uses gradients, flowing into the final convolutional layer to produce a coarse localization heatmap highlighting important regions in the image for predicting the target concept. After visual comparison of classification-based and segmentation-based approaches, we may state that visualization and decision-making based on segmentation masks are of higher quality than the localization heatmaps obtained by Grad-CAM, thus the results of the proposed two-stage workflow are more precise. This is to be expected, however, as Grad-CAM is usually used for approximate localization and requires less effort and resources for data labeling and neural network training.

## Conclusion

In this study, we proposed a workflow used for scoring and segmentation of lung diseases. The development was influenced by the standard practice adopted by trained clinicians when estimating the severity of a lung infection from an X-ray image. Central to our approach is the utilization of two core independent stages which allow us to investigate the regions of interest on an X-ray image, resulting in a lung mask and a disease mask. An additional block at the end of the workflow uses these masks to estimate the overall severity score for a given patient. To select the best solution, we compared the performance of nine neural networks in both stages. The most accurate solution, in terms of MAE and RMSE, turned out to be based on DeepLabV3 + for lung segmentation and MA-Net for disease segmentation. Having compared the solution with the state-of-the-art BS-net and COVID-Net-S, we found our proposal to be more stable in terms of accuracy and more time-efficient in terms of prediction speed.

## Supplementary Information

Supplementary Information 1.

Supplementary Information 2.

Supplementary Information 3.

Supplementary Information 4.

Supplementary Information 5.

Supplementary Information 6.

Supplementary Information 7.

## Data Availability

To study algorithm performance, we collected, cleaned, and pre-processed three lung segmentation datasets as well as four disease segmentation and scoring datasets acquired for COVID-19 and pneumonia-infected patients. The datasets are publicly available on the following Mendeley Data repositories: https://data.mendeley.com/datasets/8gf9vpkhgy/1 and https://data.mendeley.com/datasets/36fjrg9s69/1.
