# Supplementary material for: Automatic scoring of COVID-19 severity in X-ray imaging based on a novel deep learning workflow
Source: Sci Rep. 2022 Jul 27;12:12791. doi: 10.1038/s41598-022-15013-z (PMC9326426; doi:10.1038/s41598-022-15013-z)
Supplement: Supplementary file 1 — Supplementary Information 1. [file 41598_2022_15013_MOESM1_ESM.pdf]

Appendix A. Distribution of COVID-19 affected patients in the collected dataset

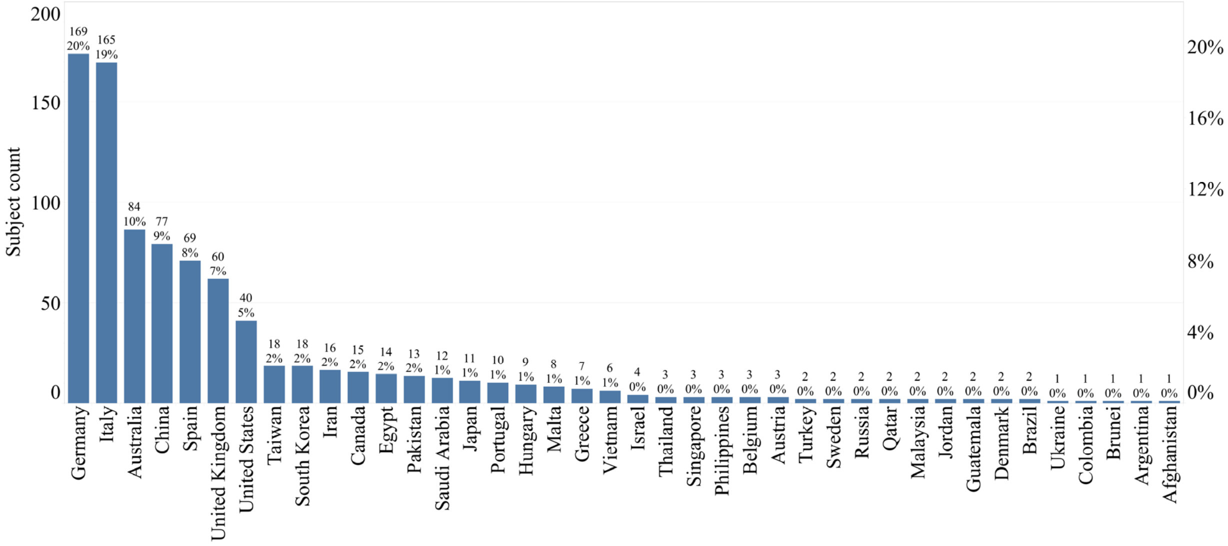

Figure A1. Representation of COVID-19 patients by country

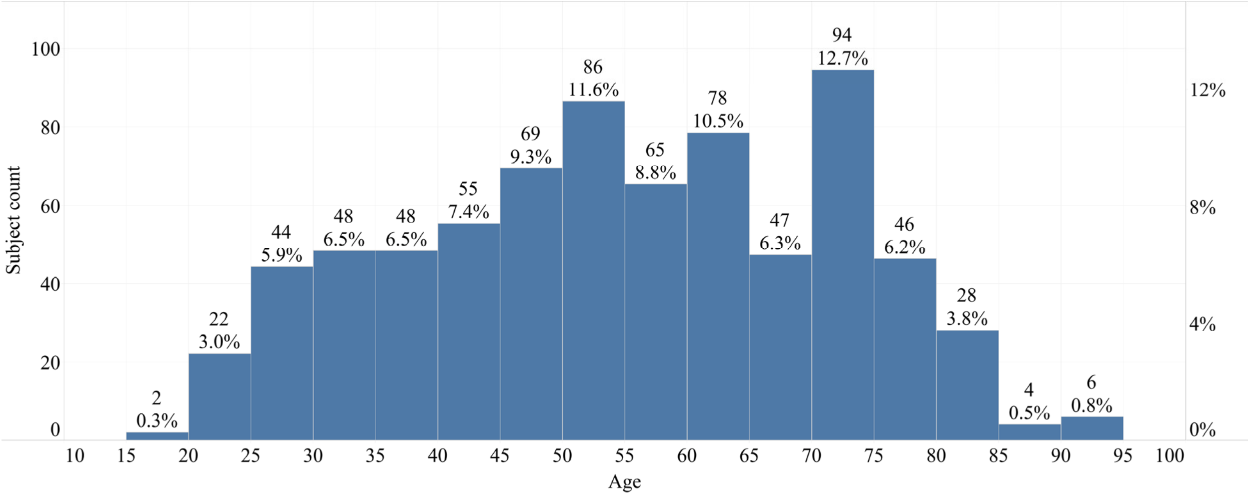

Figure A2. Representation of COVID-19 patients by age
