# Supplementary material for: Automatic scoring of COVID-19 severity in X-ray imaging based on a novel deep learning workflow
Source: Sci Rep. 2022 Jul 27;12:12791. doi: 10.1038/s41598-022-15013-z (PMC9326426; doi:10.1038/s41598-022-15013-z)
Supplement: Supplementary file 2 — Supplementary Information 2. [file 41598_2022_15013_MOESM2_ESM.pdf]

Appendix B. Pseudocode of the workflow blocks

Algorithm B1. Dynamic Loss Weighting

Input: T tasks and parameter α

Output: Re-weighted loss L_B and updated network weights W

INITIALIZE neural network weights W
FOR each epoch i **DO**
    FOR each batch B **DO**
    GET the loss on each task $L_B \in R^T$
    STORE the first batch loss as $L_{(0,i)} \in R^T$
        FOR each task t **DO**
            SET the task weight $w_t = (\frac{L_{(B,t)}}{L_{(0,i,t)}})^\alpha$
            UPDATE weighted loss $L_{(B,t)} = L_{(B,t)} \times w_t$
        END FOR
        UPDATE W with respect to L_B
    END FOR
END FOR

Algorithm B2. Segmenter

Input: Lung mask M

Output: Coordinate y_{mid} optimally splitting a lung into segments

SET h and w equal to the height and width of a lung mask M
SET a lower coordinate $y_{low} = 0$
SET an upper coordinate $y_{top} = h$
COMPUTE the desired area $S' = \frac{1}{3} \times \sum_{i=1}^{w} \sum_{j=1}^{h} M_{ij}$
WHILE $y_{low} \leq y_{top}$ **DO**
    SET a middle coordinate $y_{mid} = \frac{y_{low} + y_{top}}{2}$
    COMPUTE a segment area $A = \sum_{i=1}^{w} \sum_{j=1}^{y_{mid}} M_{ij}$
    IF $A \leq S'$ **DO**
        UPDATE the lower coordinate $y_{low} = y_{mid} + 1$
    IF $A > S'$ **DO**
        UPDATE the upper coordinate $y_{top} = y_{mid} - 1$
RETURN y_{mid}

Algorithm B3. Segment and severity score estimator

Input: Lung mask M, disease mask D, threshold T

Output: Total severity score N

SET h and w equal to the height and width of the lung mask M

INITIALIZE the sum of pixel values $S = \sum_{i=1}^{w} \sum_{j=1}^{h} M_{ij}$

RUN Algorithm 2 to divide M into three segments

SET $N = 0$

FOR each lung segment s **DO**

    SET $M_i = s \cap \mathrm{D}$

    IF $M_i \geq T$ **DO**

        UPDATE $N = N + 1$

END FOR

RETURN N
