# Supplementary material for: Automatic scoring of COVID-19 severity in X-ray imaging based on a novel deep learning workflow
Source: Sci Rep. 2022 Jul 27;12:12791. doi: 10.1038/s41598-022-15013-z (PMC9326426; doi:10.1038/s41598-022-15013-z)
Supplement: Supplementary file 3 — Supplementary Information 3. [file 41598_2022_15013_MOESM3_ESM.pdf]

Appendix C. Tuning of hyperparameters

Table C1. Hyperparameters used during the networks' optimization

**Hyperparameter**	**Value**	**Count**
Model	U-net, U-net++, DeepLabV3, DeepLabV3+, FPN, Linknet, PSPNet, PAN, MA-Net	9
Encoder	ResNet-50, ResNet-101, ResNeXt-50, ResNeXt-101, RegNetX-32, RegNetX-64, RegNetY-32, RegNetY-64, SE-ResNet-50, SE-ResNet-101, SE-ResNeXt-50, SE-ResNeXt-101, EfficientNet B0, EfficientNet B1, EfficientNet B2, MobileNet V2, SK-ResNet-34, SK-ResNet-50, DPN-68, DPN-98	20
Input size	384x384 to 640x640 with the step of 32x32 px	9
Loss (classification)	Binary cross-entropy, L1, Smooth L1	3
Loss (segmentation)	Dice, Jaccard, Binary cross-entropy, Binary cross-entropy with logits, Lovász, Focal	6
Optimizer	SGD, RMSprop, Adam, AdamW, Adam (AMSGrad), AdamW (AMSGrad)	6
Learning rate	0.01, 0.005, 0.001, 0.0005, 0.0001	5

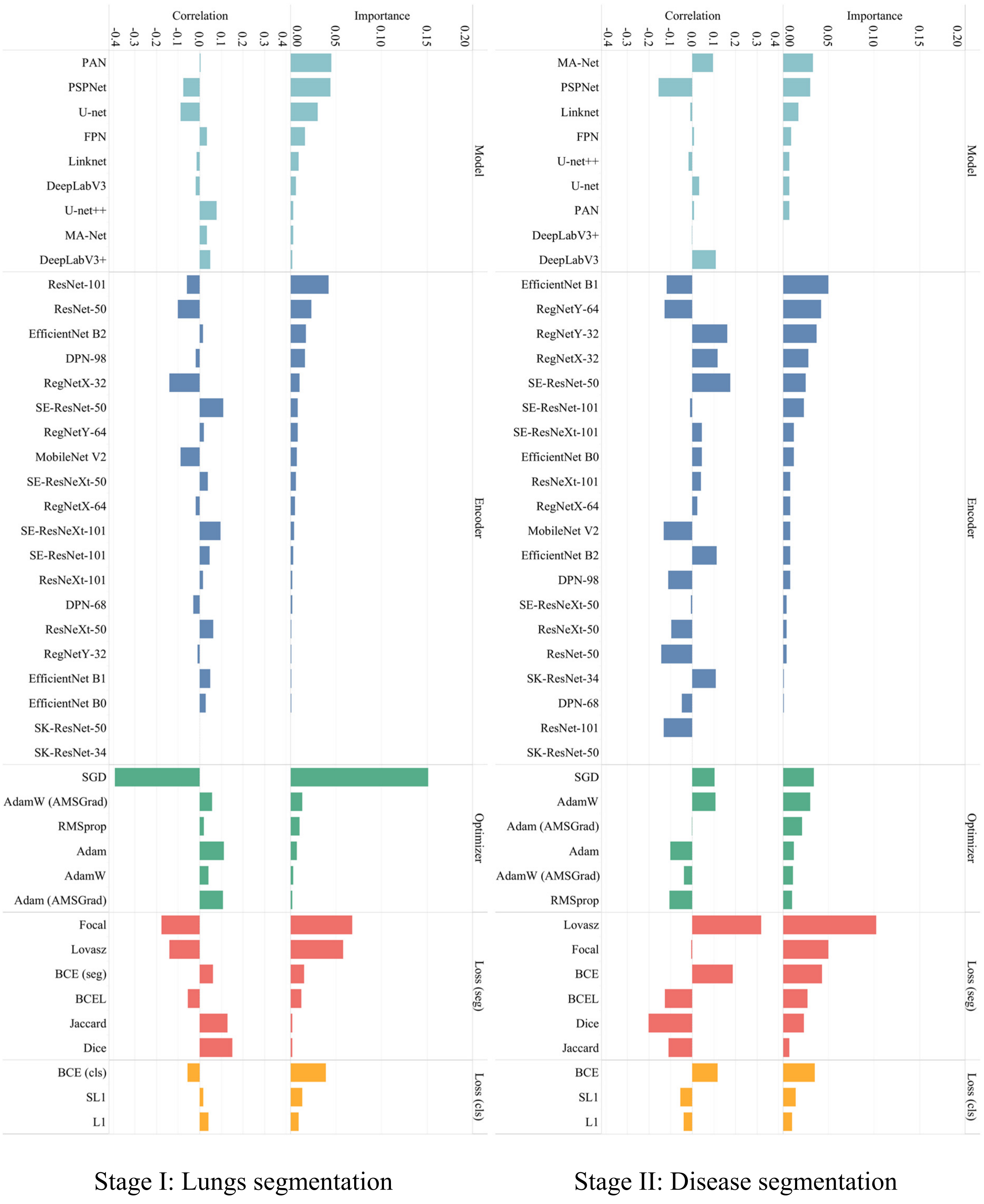

Figure C1. Correlation and importance for the tuning of networks during both stages
