# Supplementary material for: Automatic scoring of COVID-19 severity in X-ray imaging based on a novel deep learning workflow
Source: Sci Rep. 2022 Jul 27;12:12791. doi: 10.1038/s41598-022-15013-z (PMC9326426; doi:10.1038/s41598-022-15013-z)
Supplement: Supplementary file 4 — Supplementary Information 4. [file 41598_2022_15013_MOESM4_ESM.pdf]

Appendix D. Extended results for the trained, validated, and tested networks in both stages

Table D1. Results of the lung segmentation networks on Stage I

Model	Accuracy			Precision			Recall			DSC		
	Train	Val	Test	Train	Val	Test	Train	Val	Test	Train	Val	Test
U-net	0.972	0.975	0.976	0.963	0.958	0.961	0.958	0.962	0.963	0.960	0.960	0.962
U-net++	0.968	0.974	0.975	0.957	0.959	0.962	0.954	0.959	0.959	0.956	0.959	0.960
DeepLabV3	0.972	0.974	0.975	0.962	0.965	0.968	0.957	0.955	0.955	0.960	0.960	0.961
DeepLabV3+	0.973	0.975	0.976	0.965	0.965	0.969	0.958	0.956	0.958	0.962	0.960	0.963
FPN	0.974	0.974	0.975	0.965	0.958	0.962	0.959	0.962	0.961	0.962	0.960	0.962
Linknet	0.975	0.974	0.975	0.967	0.955	0.959	0.963	0.963	0.963	0.965	0.959	0.961
PSPNet	0.971	0.974	0.975	0.961	0.961	0.963	0.957	0.956	0.958	0.959	0.959	0.960
PAN	0.972	0.975	0.976	0.963	0.965	0.967	0.958	0.955	0.956	0.961	0.960	0.962
MA-Net	0.969	0.974	0.975	0.959	0.963	0.966	0.954	0.954	0.956	0.956	0.959	0.961

Table D2. Results of the COVID-19 segmentation networks (segmentation branch) in Stage II

Model	Accuracy			Precision			Recall			DSC		
	Train	Val	Test	Train	Val	Test	Train	Val	Test	Train	Val	Test
U-net	0.965	0.963	0.962	0.796	0.869	0.878	0.864	0.922	0.915	0.821	0.894	0.894
U-net++	0.964	0.963	0.955	0.787	0.723	0.714	0.875	0.870	0.845	0.825	0.724	0.702
DeepLabV3	0.892	0.903	0.911	0.966	1.000	1.000	0.021	0.462	0.500	0.016	0.462	0.500
DeepLabV3+	0.952	0.961	0.954	0.731	0.795	0.775	0.818	0.831	0.793	0.769	0.811	0.780
FPN	0.966	0.974	0.973	0.817	0.858	0.851	0.860	0.865	0.861	0.836	0.861	0.856
Linknet	0.949	0.929	0.931	0.724	0.404	0.460	0.773	0.887	0.786	0.744	0.467	0.544
PSPNet	0.945	0.965	0.962	0.677	0.863	0.726	0.818	0.876	0.826	0.738	0.869	0.771
PAN	0.939	0.941	0.944	0.727	0.536	0.652	0.615	0.593	0.616	0.660	0.349	0.606
MA-Net	0.964	0.967	0.961	0.788	0.876	0.724	0.860	0.876	0.868	0.818	0.874	0.733

Table D3. Results of the COVID-19 segmentation networks (classification branch) in Stage II

Model	Accuracy			Precision			Recall			F1		
	Train	Val	Test	Train	Val	Test	Train	Val	Test	Train	Val	Test
U-net	0.989	0.985	0.972	0.993	1.000	1.000	0.984	0.970	0.972	0.987	0.983	0.985
U-net++	0.990	0.986	0.957	0.989	0.857	0.857	0.986	0.964	0.957	0.986	0.837	0.834
DeepLabV3	0.558	0.608	0.621	0.489	1.000	1.000	0.282	0.462	0.500	0.265	0.462	0.500
DeepLabV3+	0.965	0.958	0.938	0.983	0.988	1.000	0.930	0.895	0.827	0.954	0.936	0.898
FPN	0.991	0.995	0.969	0.990	1.000	0.963	0.988	0.989	0.970	0.989	0.994	0.966
Linknet	0.940	0.811	0.876	0.945	0.542	0.690	0.908	0.993	0.993	0.924	0.586	0.708
PSPNet	0.964	0.948	0.938	0.958	1.000	1.000	0.952	0.902	0.885	0.954	0.946	0.935
PAN	0.902	0.854	0.877	0.911	0.659	0.644	0.846	0.912	0.965	0.872	0.615	0.678
MA-Net	0.984	0.993	0.934	0.981	1.000	0.859	0.978	0.969	0.922	0.978	0.982	0.824
