# Supplementary material for: Automatic scoring of COVID-19 severity in X-ray imaging based on a novel deep learning workflow
Source: Sci Rep. 2022 Jul 27;12:12791. doi: 10.1038/s41598-022-15013-z (PMC9326426; doi:10.1038/s41598-022-15013-z)
Supplement: Supplementary file 5 — Supplementary Information 5. [file 41598_2022_15013_MOESM5_ESM.pdf]

Appendix E. Scoring performance estimated separately on COVID-19 and normal datasets

Table E1. Scoring performance estimated on the dataset of COVID-19 cases

Stage I model	Stage II model	MAE			RMSE		
		Training	Validation	Testing	Training	Validation	Testing
DeepLabV3+	U-net	0.75	0.79	0.80	1.03	1.04	1.06
	U-net++	1.01	1.00	0.95	1.45	1.31	1.39
	DeepLabV3	3.84	3.96	3.62	4.23	4.35	3.99
	DeepLabV3+	0.99	1.08	0.89	1.34	1.50	1.13
	FPN	0.76	0.83	0.73	1.05	1.08	1.05
	Linknet	0.92	1.06	0.89	1.24	1.45	1.26
	PSPNet	1.05	0.92	0.87	1.43	1.31	1.30
	PAN	1.11	1.06	1.18	1.53	1.32	1.65
	MA-Net	0.83	0.83	0.69	1.12	1.09	0.99
BS-net		1.18	1.00	1.45	1.63	1.35	1.85
COVID-Net-S		1.23	1.27	1.29	1.44	1.44	1.52

Table E2. Scoring performance estimated on the dataset of normal cases

Stage I model	Stage II model	MAE			RMSE		
		Training	Validation	Testing	Training	Validation	Testing
DeepLabV3+	U-net	0.00	0.00	0.00	0.00	0.00	0.00
	U-net++	0.00	0.00	0.03	0.00	0.00	0.22
	DeepLabV3	0.00	0.00	0.00	0.00	0.00	0.00
	DeepLabV3+	0.00	0.00	0.00	0.04	0.00	0.00
	FPN	0.02	0.01	0.10	0.17	0.11	0.50
	Linknet	0.01	0.10	0.03	0.17	0.58	0.22
	PSPNet	0.04	0.09	0.04	0.26	0.38	0.25
	PAN	0.15	0.17	0.09	0.60	0.67	0.43
	MA-Net	0.00	0.00	0.03	0.00	0.00	0.22
BS-net		3.15	3.09	3.25	3.66	3.61	3.76
COVID-Net-S		2.21	2.18	2.20	2.35	2.33	2.36
