# Supplementary material for: Automatic scoring of COVID-19 severity in X-ray imaging based on a novel deep learning workflow
Source: Sci Rep. 2022 Jul 27;12:12791. doi: 10.1038/s41598-022-15013-z (PMC9326426; doi:10.1038/s41598-022-15013-z)
Supplement: Supplementary file 6 — Supplementary Information 6. [file 41598_2022_15013_MOESM6_ESM.pdf]

Appendix F. Visualization of the probability maps for the COVID-19 datasets

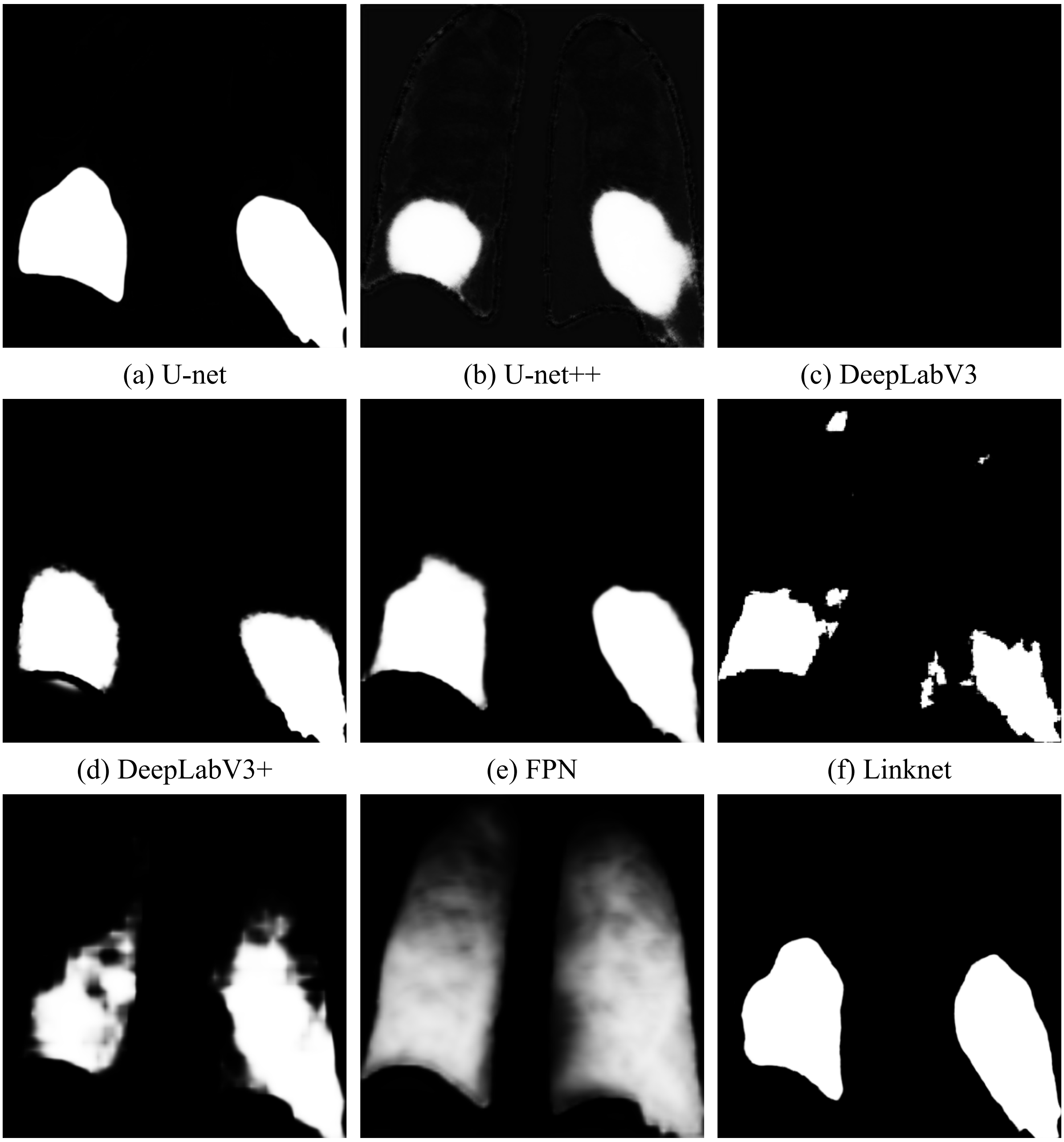

(a) U-net (b) U-net++ (c) DeepLabV3

(d) DeepLabV3+ (e) FPN (f) Linknet

(g) PSPNet (h) PAN (i) MA-Net

Figure F1. Comparison of the probability maps of a COVID-19 subject from the CRD dataset

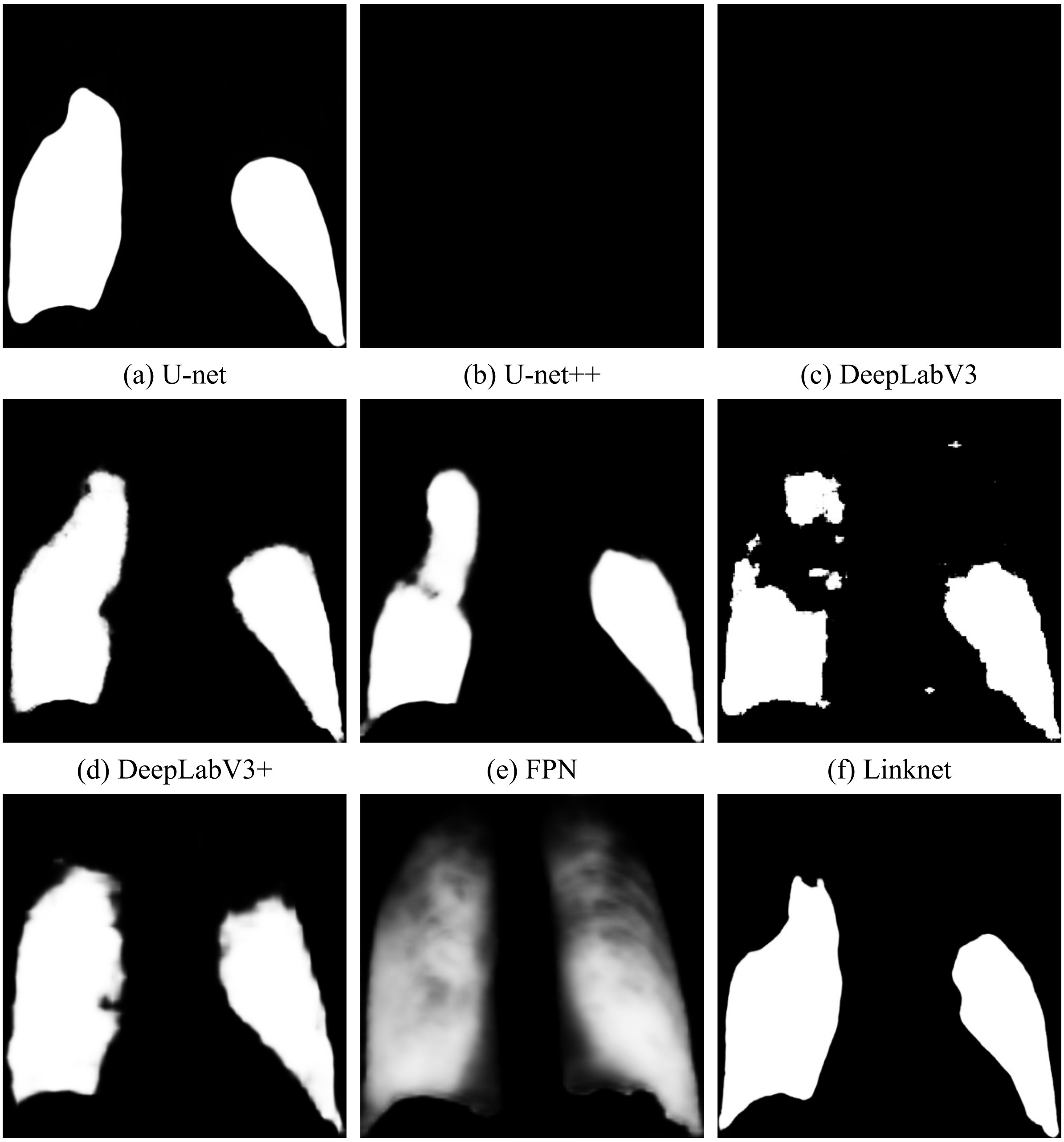

(g) PSPNet (h) PAN (i) MA-Net

Figure F2. Comparison of the probability maps of a COVID-19 subject from the CCXD dataset

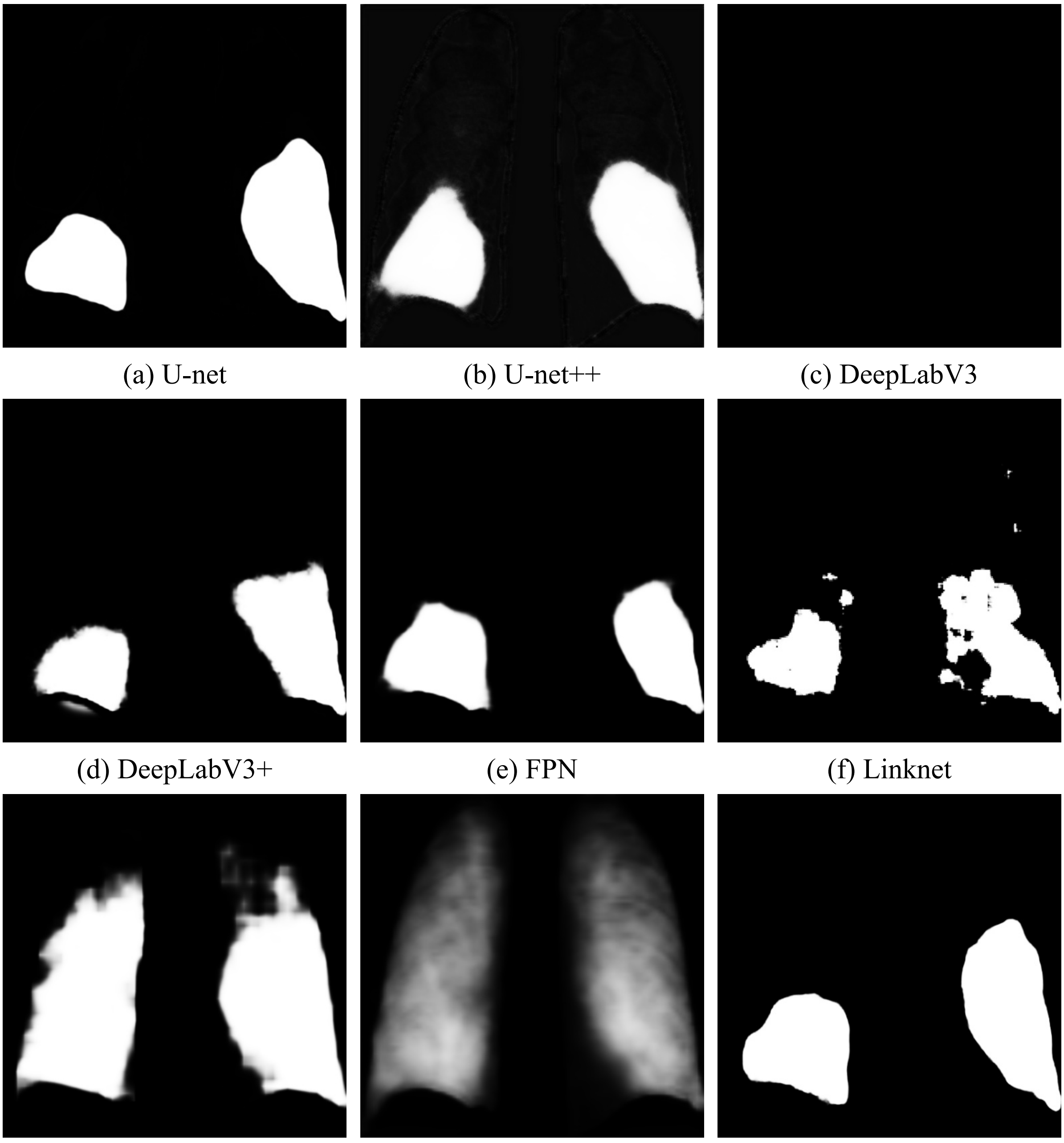

Figure F3. Comparison of the probability maps of a COVID-19 subject from the FCXD dataset
