# Supplementary material for: Automatic scoring of COVID-19 severity in X-ray imaging based on a novel deep learning workflow
Source: Sci Rep. 2022 Jul 27;12:12791. doi: 10.1038/s41598-022-15013-z (PMC9326426; doi:10.1038/s41598-022-15013-z)
Supplement: Supplementary file 7 — Supplementary Information 7. [file 41598_2022_15013_MOESM7_ESM.pdf]

Appendix G. Visualization of the segmentation and scoring for COVID-19 datasets

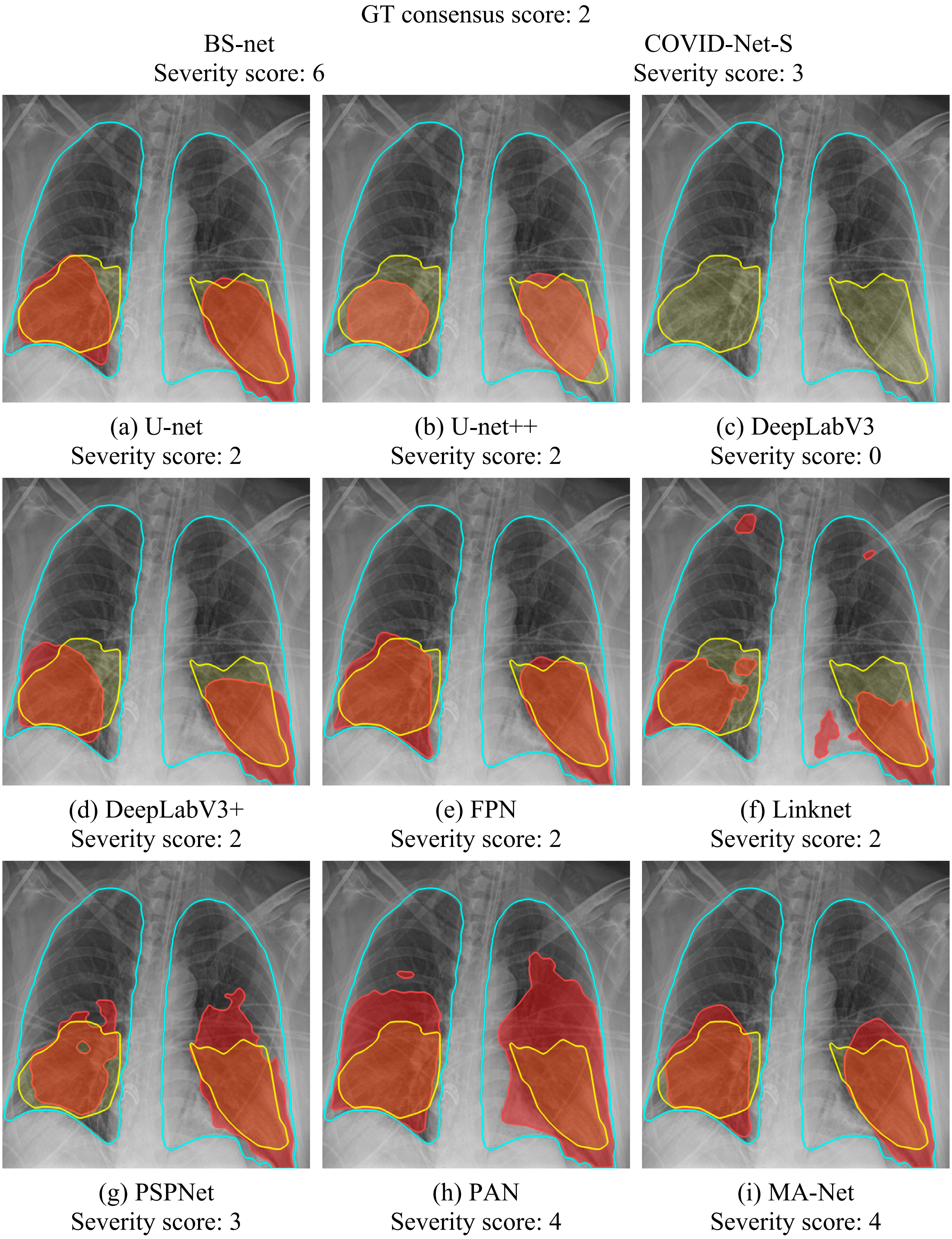

Figure G1. Comparison of the segmentation and severity score estimation of a COVID-19 subject from the CRD dataset. A cyan delineation refers to the lung segmentation obtained by Stage I; a red mask is a disease mask obtained by Stage II; a yellow mask refers to the ground-truth segmentation of the disease

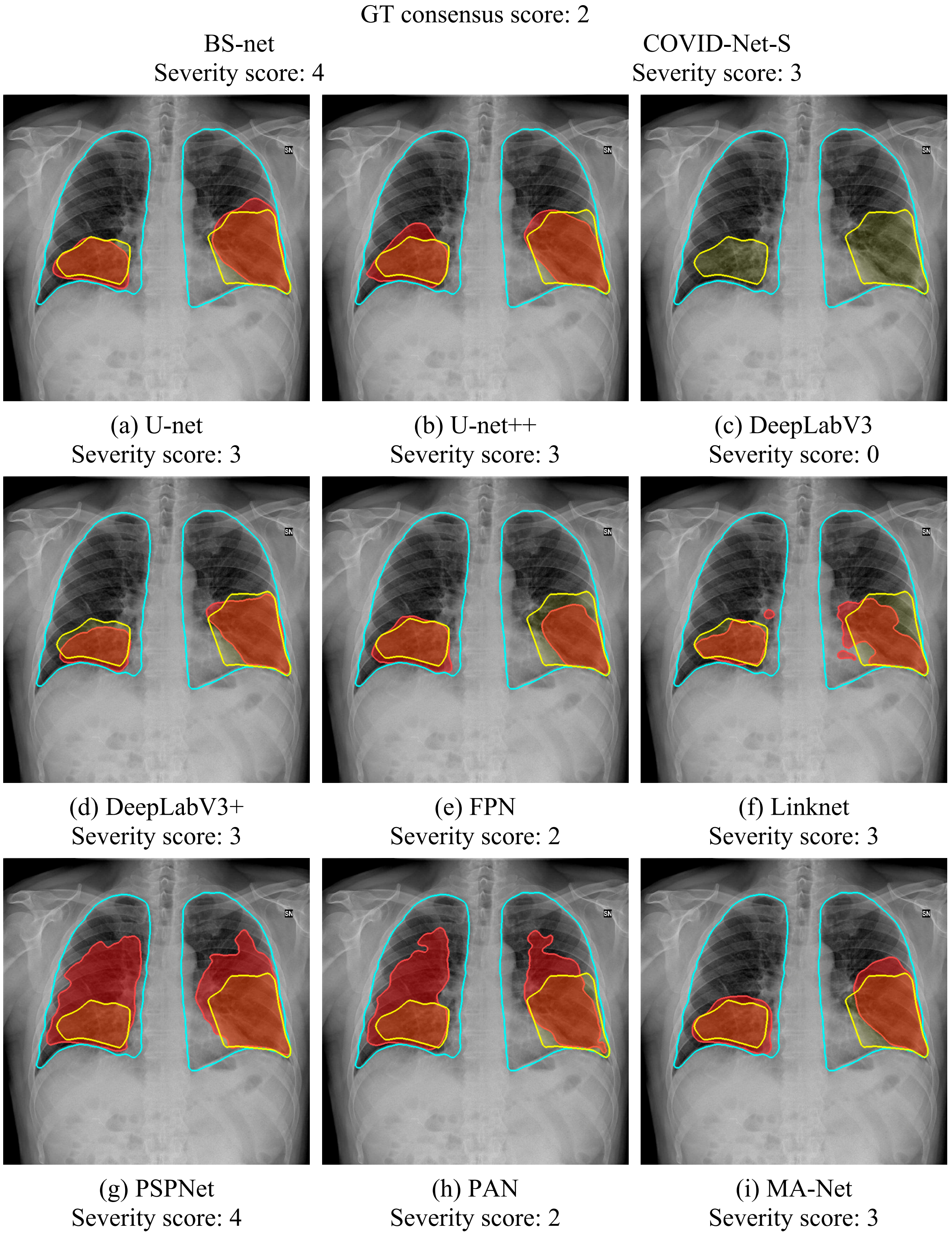

Figure G2. Comparison of the segmentation and severity score estimation of a COVID-19 subject from the FCXD dataset. A cyan delineation refers to the lung segmentation obtained by Stage I; a red mask is a disease mask obtained by Stage II; a yellow mask refers to the ground-truth segmentation of the disease

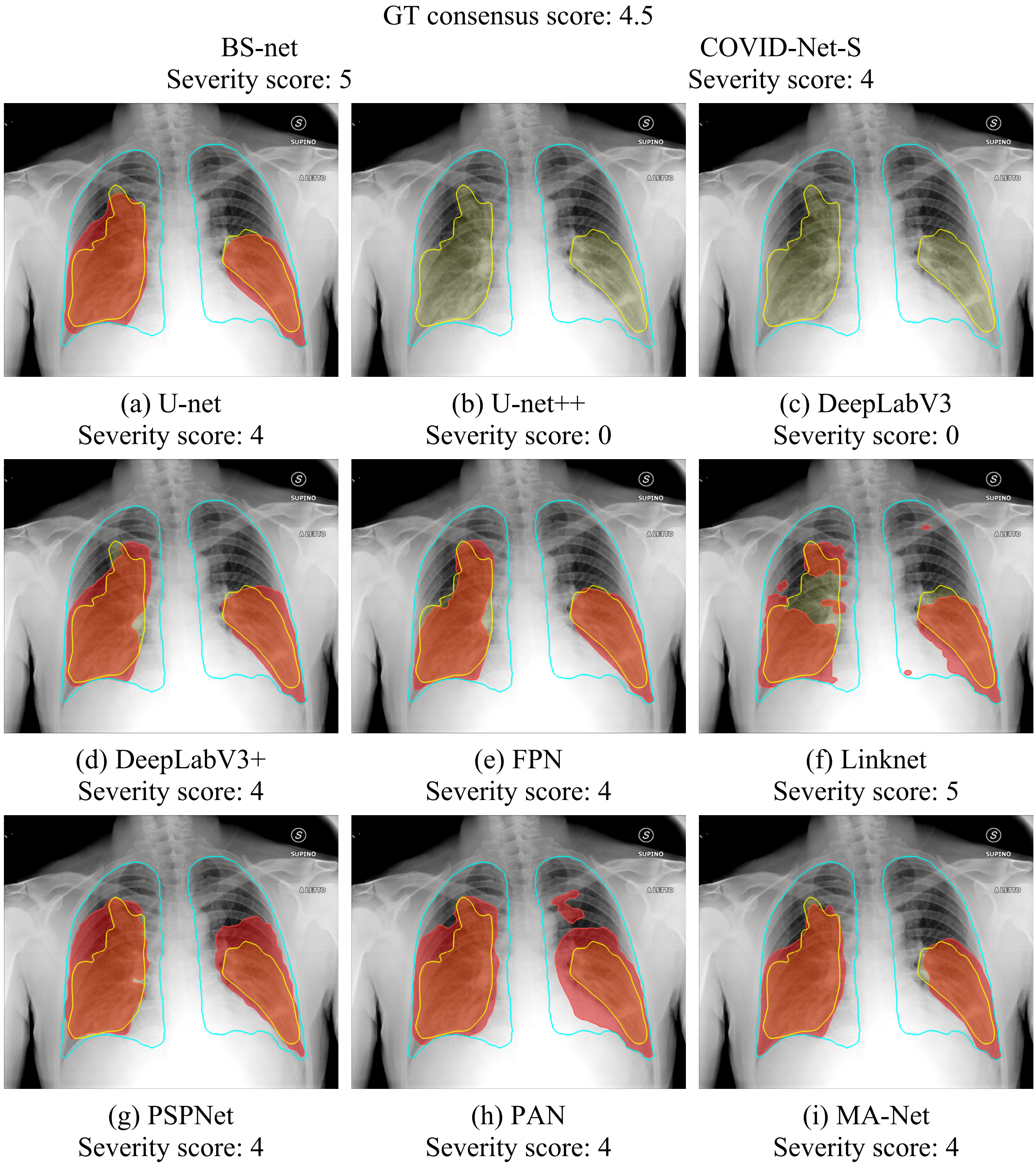

Figure G3. Comparison of the segmentation and severity score estimation of a COVID-19 subject from the CCXD dataset. A cyan delineation refers to the lung segmentation obtained by Stage I; a red mask is a disease mask obtained by Stage II; a yellow mask refers to the ground-truth segmentation of the disease
